# Watching Molecular Nanotubes Self-Assemble in Real Time

**DOI:** 10.1021/jacs.3c07103

**Published:** 2023-10-06

**Authors:** Marìck Manrho, Sundar Raj Krishnaswamy, Björn Kriete, Ilias Patmanidis, Alex H. de Vries, Siewert J. Marrink, Thomas L. C. Jansen, Jasper Knoester, Maxim S. Pshenichnikov

**Affiliations:** † Zernike Institute for Advanced Materials, University of Groningen, Nijenborgh 4, 9747 AG Groningen, The Netherlands; ‡ Groningen Biomolecular Sciences and Biothechnology Institute, University of Groningen, Nijenborgh 7, 9747 AG Groningen, The Netherlands; § Department of Chemistry, Aarhus University, Langelandsgade 140, 8000 Aarhus C, Denmark; ∥ Faculty of Science, Leiden University, Einsteinweg 55, 2300 RA Leiden, The Netherlands

## Abstract

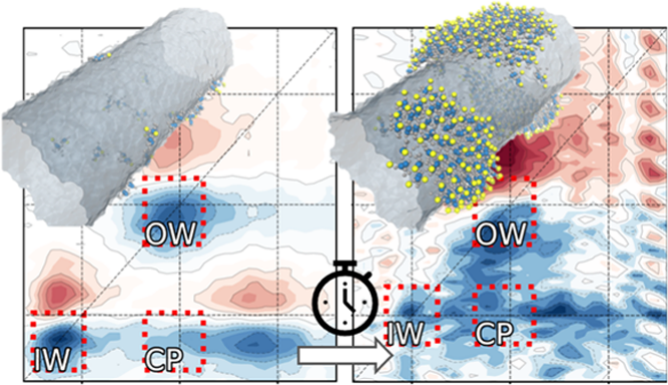

Molecular self-assembly is a fundamental process in nature that can be used to develop novel functional materials for medical and engineering applications. However, their complex mechanisms make the short-lived stages of self-assembly processes extremely hard to reveal. In this article, we track the self-assembly process of a benchmark system, double-walled molecular nanotubes, whose structure is similar to that found in biological and synthetic systems. We selectively dissolved the outer wall of the double-walled system and used the inner wall as a template for the self-reassembly of the outer wall. The reassembly kinetics were followed in real time using a combination of microfluidics, spectroscopy, cryogenic transmission electron microscopy, molecular dynamics simulations, and exciton modeling. We found that the outer wall self-assembles through a transient disordered patchwork structure: first, several patches of different orientations are formed, and only on a longer time scale will the patches interact with each other and assume their final preferred global orientation. The understanding of patch formation and patch reorientation marks a crucial step toward steering self-assembly processes and subsequent material engineering.

## 1 Introduction

Molecular self-assembly is one of the pillars in the fabrication of functional (bio)chemical systems, with examples ranging from liquid crystals^1,2^ and light-harvesting complexes^3,4^ to protein-based materials.^5,6^ In molecular self-assembly processes, the generation of supramolecular structures is driven by a wide variety of interactions such as van der Waals forces, π–π stacking,^7^ halogen and hydrogen bonding,^8−12^ and hydrophobic and ionic interactions.^13,14^ Molecular self-assembly generally occurs within solutions, resulting in molecular systems with relatively large structural disorder (both static and dynamic) compared to covalently bonded crystals.^15,16^ Furthermore, the self-assembly of molecular complexes takes place on a wide range of time scales ranging from nanoseconds up to hours.^17,18^ The high degree of molecular disorder as well as the broad distribution of time scales obstruct the successful use of conventional X-ray or electron-based imaging to obtain a detailed molecular view of the self-assembly processes. Nevertheless, understanding the mechanisms that govern the self-assembling process in such systems throughout the entire complex of hierarchical structures, from individual molecules, over individual subunits all the way up to the complete assembly, is vital to unravel nature’s highly successful design principles.

This research provides a new platform that allows studying of molecular self-assembly processes of supramolecular structures in real time. A system that lends itself to this study is the C8S3^1^-based double-walled nanotube (DWNT), the vast interest in which is driven by its close resemblance to the light-harvesting antennae in green sulfur bacteria,^4,19^ with potential applications as a long-range energy transport wire in photovoltaics.^20,21^ The C8S3 DWNT has an inner (outer) diameter of ∼7 nm (∼14 nm) with lengths extending several microns^22,23^ and consists of hundreds of thousands of tightly packed dye molecules.^24−26^ This system is uniquely suited for a controlled study of self-assembly as its outer wall can be readily removed by breaking the molecular interactions between the inner and outer walls through the addition of a water/methanol mixture (the process known as flash-dilution^22,27^), leaving the intact inner wall as a template for subsequent reassembly of the outer wall. Furthermore, as the DWNT consists of highly absorbing/fluorescing dye molecules, the reassembly process can be conveniently tracked by optical spectroscopy, which provides access to fleeting reassembly stages. Notably, comprehensive understanding of the DWNT system accumulated over the past decades through (nonlinear) spectroscopy,^26,28−30^ molecular dynamics,^17,31−33^ exciton modeling,^25,32^ and very recently near-atomic resolution cryo-TEM imaging^23^ serves as an excellent reference point in the self-(re)assembly process.

Here, we show that the combination of microfluidics, cryo-TEM imaging, ultrafast correlation spectroscopy, and linear-dichroism spectroscopy provides real-time information about the self-assembly process. Furthermore, by combining the abovementioned experimental techniques with molecular dynamics simulations and exciton modeling, a detailed picture of the self-assembly processes is drawn from molecules to a supramolecular structure. We demonstrate that the self-assembly occurs in two stages: first, surface-adhered relatively small molecular structures are formed that are ordered internally but are disordered with respect to each other; and later, these structures are rearranged to form the final well-ordered supramolecular double-walled structure. Our findings form a key step in understanding the self-assembly stages that could be utilized for further material design and fabrication.

## 2 Results

### 2.1 Outer-Wall Self-Assembly after Microfluidic Flash-Dilution

**Figure 1:**
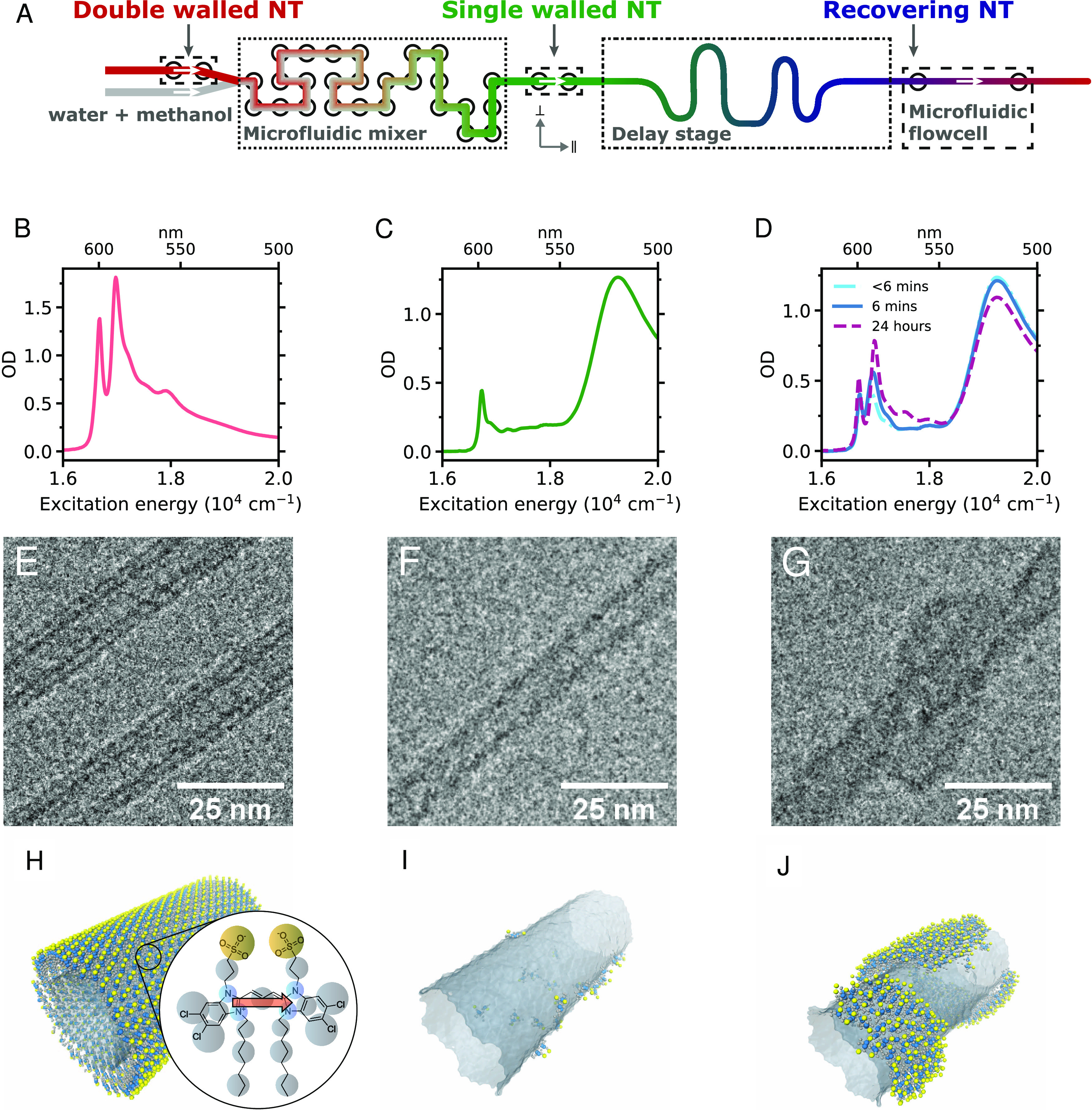
Different stages of the self-assembly experiment. (A) Schematic diagram of the three different stages in the microfluidic setup. DWNT solution and a mixture of water and methanol enter the microfluidic mixer (···), where the OW is selectively dissolved, resulting in isolated IWs and dissolved monomers. During the delay stage (·−), the isolated IWs recover into DWNTs. The self-assembly process is measured at different stages in a microfluidic flow cell or a cuvette (−). (B–D) Isotropic absorption spectra (measured in a cuvette) of DWNTs, isolated IWs, and IWs with partial OW recovery at different times after flash-dilution, respectively. (E–G) Cryo-TEM images at the three different stages showing a DWNT, an IW, and an IW with a partially recovered OW consisting of patches, respectively. (H–J) Snapshots of a coarse-grained MD trajectory showing similar stages of the flash-dilution process as captured in the cryo-TEM images. The IW is shown as a gray semitransparent surface. The molecules constituting the (recovering) OW are shown individually. The inset in panel (H) displays the chemical structure and coarse-grained model of the C8S3 building blocks,^36^ while the red arrow indicates the transition dipole moment of the chromophore core.^32^

The self-assembly process of DWNTs from individual C8S3 molecules is challenging to study due to several possible intermediate structures^34^ that are short-lived nonequilibrium states. Therefore, we restrict the self-assembly process, where DWNTs were first brought into a nonequilibrium state by removing the outer wall with flash-dilution,^3^ following which, the subsequent reformation of the outer wall occurs with the inner wall acting as a template. Flash-dilution was carried out in a microfluidic setting^22,27^ (Figure 1A) by mixing the DWNT solution with a mixture of methanol and water, which resulted in selective dissolution of the outer wall (OW), leaving behind an inner wall (IW) with hydrophobic tails exposed to the aqueous surrounding (see Section 4.2). Thereafter, the reassembly of the outer wall could be monitored in real time by optical spectroscopy (absorption, linear dichroism, and 2D) as it reached a new equilibrium and with snapshots at representative intervals using cryo-TEM.

Prior to microfluidic flash-dilution, C8S3 DWNTs are characterized by two prominent narrow absorption peaks at ∼17,000 and ∼16,700 cm^–1^ (Figure 1B), which are associated with exciton transitions in the OWs and IWs, respectively,^10,32^ and are polarized parallel to the long tubular axis.^35^ Meanwhile, higher-lying transitions >17,200 cm^–1^ have overlapping contributions from both walls and contain both parallel and perpendicularly polarized transitions.^23,35^ Cryo-TEM imaging showed that the DWNTs have outer and inner diameters of 13.5 ± 0.4 and 6.9 ± 0.3 nm, respectively, and tube lengths up to several μm, which is consistent with previous results.^10,22^

Successful flash-dilution is evident from the absence of the OW absorption peak at ∼17,000 cm^–1^ (Figure 1C). The low-energy IW transition (∼16,700 cm^–1^) after flash-dilution retains its polarization along the length of the NT, which agrees well with an earlier finding on the preservation of the IW structure after flash-dilution.^22^ Additionally, a broad unpolarized peak centered at ∼19,100 cm^–1^ appears, which corresponds to the absorption spectrum of the dissolved C8S3 molecules.^3,26,27^

After flash-dilution, C8S3 monomers (i.e., the C8S3 molecules from the former OWs and partially IWs dissolved in a methanol/water mixture) are adsorbed onto the IWs, causing the recovery of the OWs. The reformation takes place as the flash-diluted C8S3 monomers travel out of the microfluidic mixer to the flow cell (Figure 1A), which allows spatial separation of the region where IWs are produced from the region where reformation of OWs starts. The recovery of the OWs occurs on a time scale of several tens of seconds up to minutes and hours and is observed from an ingrowing absorption peak at ∼17,000 cm^–1^ (Figures 1D and S1). The intact IW^22^ acts as a template for the reforming OW, which enables the use of cryo-TEM and polarization-sensitive spectroscopy techniques to unravel the reassembly process of the dissolved monomers.

### 2.2 TEM and MD Provide Snapshots of the Self-Assembly Process

Different stages of the reassembly process after flash-dilution were monitored by cryo-TEM (for details, see Section 4.3). The cryo-TEM images shown in Figure 1E–G are depicted together with different snapshots of a coarse grain (CG) MD simulation (Figure 1H–J), which display similar structural configurations (see also Section 4.4). Before flash-dilution (Figure 1E,H), the NT is double-walled as indicated in the TEM image by a total of four contrast lines per NT, where the two inner and outer contrast lines correspond to the IW and OW.^10,22^ Right after flash-dilution (Figure 1F,I), only the IW remains intact, as indicated by the two inner contrast lines per NT.^22^ The partially recovered NTs (Figure 1G,J) display the formation of patches, which is confirmed by direct comparison of measured and simulated TEM-image line profiles (Section S2). While TEM gives a snapshot of the overall shape of the recovering OW at one particular time, dynamics of its molecular structure can only be retrieved by more advanced spectroscopic techniques and corresponding modeling.

### 2.3 Transient Linear-Dichroism Probes Different Stages of the Self-Assembly Process

**Figure 2:**
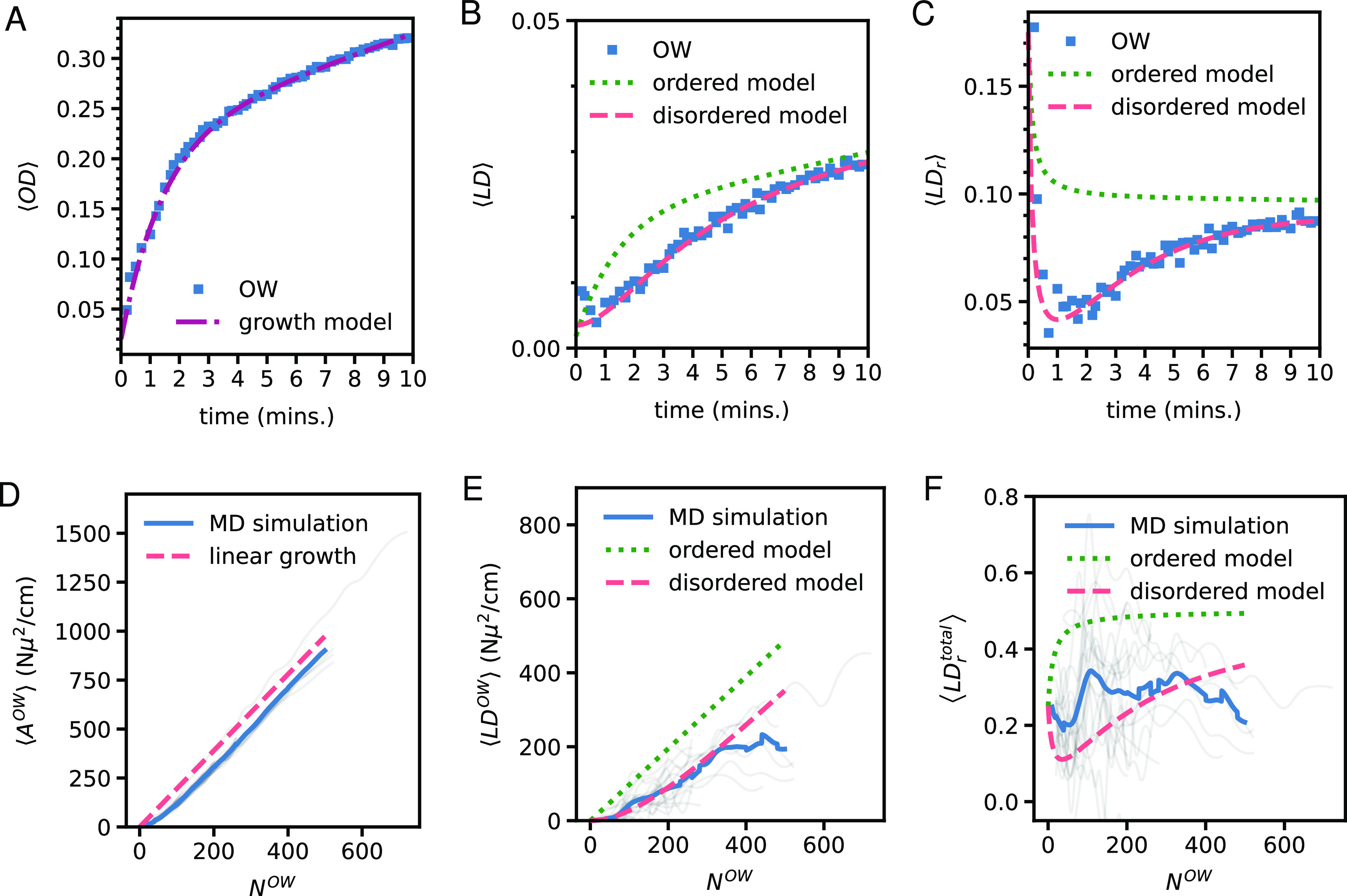
Optical density (A, D), linear dichroism (B, E), and reduced linear dichroism (C, F) of recovering nanotubes as a function of recovery time (in the case of the simulated data in the bottom row, measured in the number of added outer-wall molecules). The upper and lower rows show experimental data (blue squares) and the results of MD simulations through exciton modeling, respectively. The spectral region of the OW peak (16,892–17,094 cm^–1^) was considered in panels (A–C), over which the data are averaged; for MD simulations (D–F), an average over 15,324–18,550 cm^–1^ was taken. The different simulations, which vary in length (see SI S6 for more details), are shown by the gray lines with the average being the blue line. The results from the rate equation models are shown by the dotted green (immediate molecular ordering upon self-assembly) and dashed red (delayed ordering) lines. Note that in both experimental and MD simulated data, the initial growth of the LD signal is slower than the growth in the optical density, causing a decreasing LD_r_.

The dynamics of the OW self-assembly process was investigated by monitoring the orientation of transition dipole moments in the recovering OW at different times after flash-dilution in a microfluidic setting. The NTs align with the flow direction due to their large aspect ratio,^27^ which enables obtaining parallel and perpendicular (with respect to the sample flow direction) polarized absorption spectra. Figure 2A–C shows the resulting optical density, linear dichroism (LD), and the ratio between linear dichroism and optical density (reduced linear dichroism, LD_r_) in the OW spectral region (16,892–17,094 cm^–1^) at different times after flash-dilution. The equations used to calculate LD_*r*_ and from the polarized absorption spectra are given in Section S3.

The optical density (Figure 2A) grows rapidly in the first two minutes after flash-dilution, which is attributed to the self-assembly of the dissolved molecules on or around the isolated IWs, resulting in the reformation of OWs. In contrast, the dynamics of the LD signal (Figure 2B) is much slower. For the LD signal to grow, not only there must be formation of OWs but also the transition dipole moments of molecules that constitute the self-assembling OWs must align macroscopically in a preferred direction—which apparently is not the case in the first minute. Hence, the OW first self-assembles in disordered isotropic structures, due to which the LD_r_ value (Figure 2C) decreases in the first minute after flash-dilution. It is only after ∼2 min when the randomly oriented molecules of the recovering OW start to align into an ordered anisotropic configuration, causing an increase in LD and LD_r_ values. We note that similar data obtained from the spectral region of the IW, which is unaffected by flash-dilution and therefore can serve as a reference, show entirely different trends (Figure S5).

The growth of OD, LD, and LD_r_ is described by empirical rate equation models (see Section S4). As the OW reforms, it becomes harder for new monomers to adsorb to the OW, leading to an inverse exponential growth of the OD (red line in Figure 2A). When new molecules adsorb to the OW, they can directly align with the ordered macroscopically preferred direction, or first take some intermediate disordered orientation before macroscopic alignment occurs. In the former (ordered) case, the LD is directly proportional to the OD (green dotted line in Figure 2B) causing the LD_r_ to increase monotonically over time (green dotted line in Figure 2C), which fails to match the experimental data. In contrast, the latter (disordered) case leads to a delayed growth of LD with respect to the OD (red dashed line in Figure 2B), due to which LD_r_ decreases in the first minute, but then starts to increase (red dashed line in Figure 2C). The disordered model fits the experimental data almost perfectly.

### 2.4 Multiscale Modeling Captures Early Stages of Self-Assembly

To study the early steps of the OW self-assembly, we performed coarse-grained molecular dynamics (CG-MD) simulations using a recently developed CG model,^34^ based on the Martini 3 force field.^37^ CG simulations based on Martini have been previously used to successfully describe supramolecular self-assembly for a wide range of systems.^38,39^ In the simulations, we mimicked the experimental conditions after flash-dilution by titration. Specifically, we constructed an IW segment and placed a small number of C8S3 monomers at random positions in the solution surrounding the IW, starting the production phase. At regular intervals, we added another batch of monomers to the simulation box: we thus titrated the IW segment with fresh C8S3 monomers in a number of steps. Inspired by recent theory on the narrow escape problem,^40^ we also performed random walk simulations assuming a concentration of the monomers similar to that under experimental conditions, but homogeneously distributed in the reaction volume. We found that, on average, the IW is fully covered by the monomers no faster than that on the millisecond time scale, which by far exceeds our computational resources. Therefore, we chose to run multiple independent titration simulations at a higher addition rate rather than a single one at a lower addition rate. For more details of the simulation model and settings, see Sections 4.4 and S6, S7.

**Figure 3:**
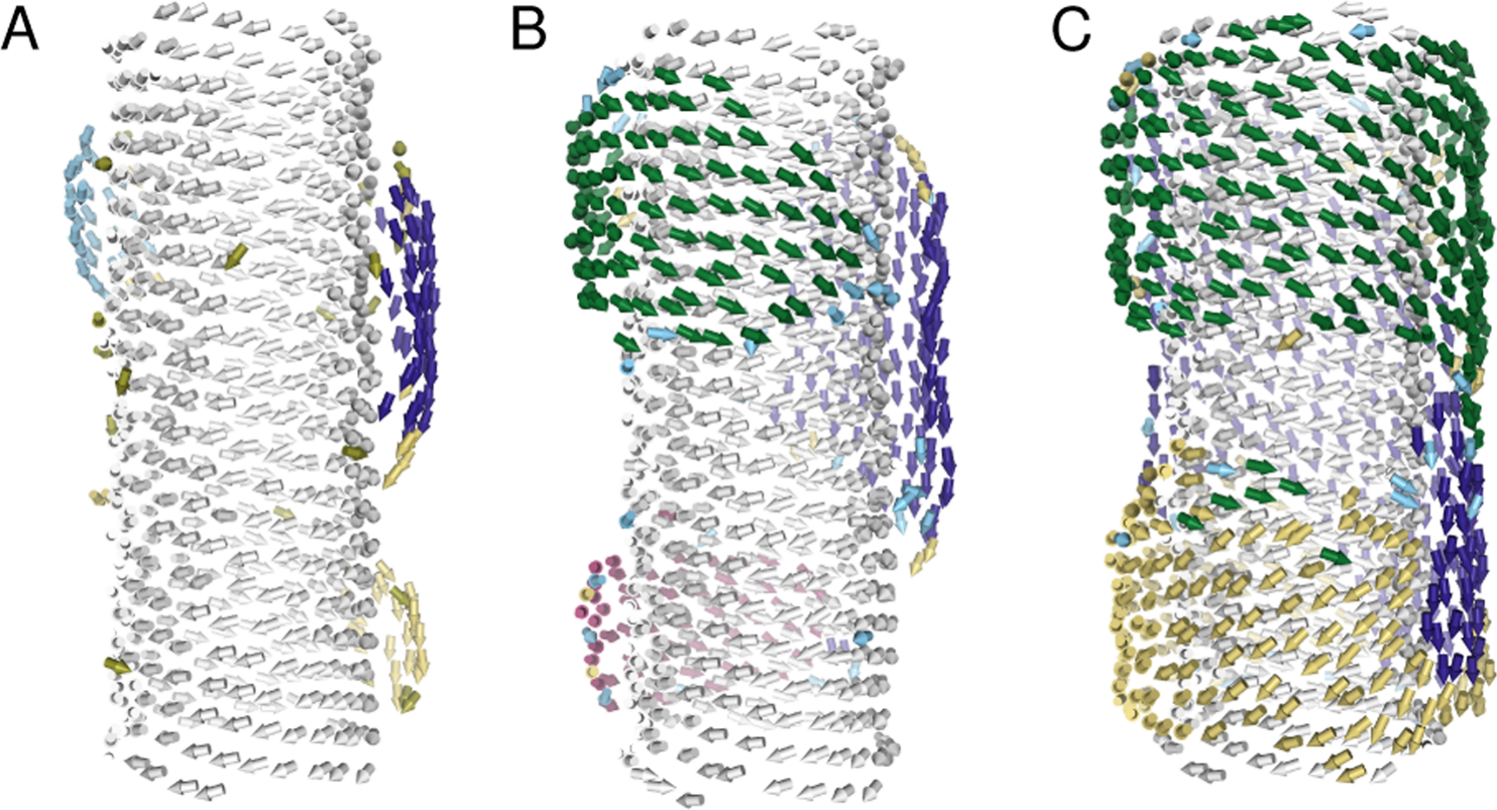
Snapshots of a CG-MD simulation of 100 (A), 300 (B), and 780 (C) C8S3 molecules, showing dynamic patches in the OW on a 20 nm segment of IW. Each C8S3 molecule is represented by an arrow that points in the direction of its transition dipole moment. The color of the arrow reflects the patch to which the molecule is assigned (see Section 4.5 for more details).

The titration simulations allowed us to witness the gradual arrival of monomers on the IW and the reassembly of the OW, from monomers to patches on the surface of the IW. During the titration simulations, the OW was partially recovered, since the dissolved monomers migrated toward the solvent-exposed hydrophobic tails of the IW. Most of the added monomers adsorb on the IW before a fresh batch is added. The adsorbed molecules move around on the IW surface and rotate to form patches of recovering OW (see Figure 3). Within each patch, the molecules are locally ordered into a brickwork structure, consistent with the recently resolved experimental packing motif.^23^ Depending on its size, an isolated patch can move and rotate on the surface of the IW, until it encounters other patches. As more monomers adsorb to the IW, the OW recovers as a patchwork. The patches merge into several large domains that cover the circumference of the inner tube. None of the titration simulations resulted in the formation of a single domain. Instead, when the largest patches meet each other, they keep their own orientation, resulting in domain walls as illustrated in Figure 3C. The recovery of the experimental LD_r_ signal in Figure 2C indicates that these domain walls will eventually vanish over the course of >2 min. This observation suggests that the process of patch and domain formation may be comparable to island growth in monolayer materials.^41,42^ An animation of a CG-MD titration simulation showing patch formation and patch reorientation can be found in the Supporting Information.

In an attempt to gain more insight into the mechanism of the reforming OW, we have performed a number of long simulations at an elevated temperature of 400 K in which we study a constant number of molecules on a partly covered 20 nm tube (i.e., no molecules are added to the system meanwhile). We observe that a number of processes take place, such as individual molecules migrating on the surface, small patches of molecules migrating and reorienting on the surface, and individual molecules attaching to and detaching from patches or domains. Thus, the processes that are typical for nucleation and growth mechanisms but also isodesmic processes or domain wall/grain boundary healing processes are all observed. At the moment, the statistics is insufficient to clearly quantify the importance or contribution of these processes, especially at 300 K for which we would need orders of magnitude longer simulations due to strong interactions between the molecules in the IW and OW and even stronger interactions between molecules in the reforming OW. An overview of the types of interactions that play a role in this system is given in Figure S6. We leave a more detailed discussion on this issue for a forthcoming publication.

The MD titration simulations were combined with exciton modeling^25,32,33,35,43,44^ to predict the spectroscopic response of the domains in the self-assembling OWs (Figure 2). The positions and orientations of the C8S3 molecules were extracted at the end of each titration step of the MD simulation. The C8S3 molecules were modeled as two-level systems that interact with each other through resonant excitation energy transfer interactions of dipolar origin. An example of the resulting absorption spectra can be found in Figure S10. The individual patches have a red-shifted absorption spectra, while interactions between separate patches have a small impact on the absorption spectra as shown in Figure S11. The calculated spectra were treated like the experimental spectra to obtain OD, LD, and LD_r_ signals as a function of the number of molecules in the OW, which are shown in Figure 2D–F. A slightly larger wavenumber window was considered for the simulated spectra to average over the entire simulated OW spectrum. At the early stages of MD simulations, OW molecules are very mobile and can briefly form small structures that do not (yet) display any significant spectroscopic red shift. This causes the spectroscopic response of the MD simulations to lie below the linear model line in Figure 2D. This also explains why the total LD_r_ of the MD simulations lies between the ordered and disordered models in the beginning of the simulation. The calculated signals are fitted best with the disordered model, suggesting that the transient disordered state observed in the experiment is due to the OW self-assembling as a patchwork. Further details of the simulated absorption and linear-dichroism spectra are found in Section 4.6.

### 2.5 2D Visible Spectroscopy (2D vis) Spectroscopy Confirms a Patch-Like Growth Mechanism

**Figure 4:**
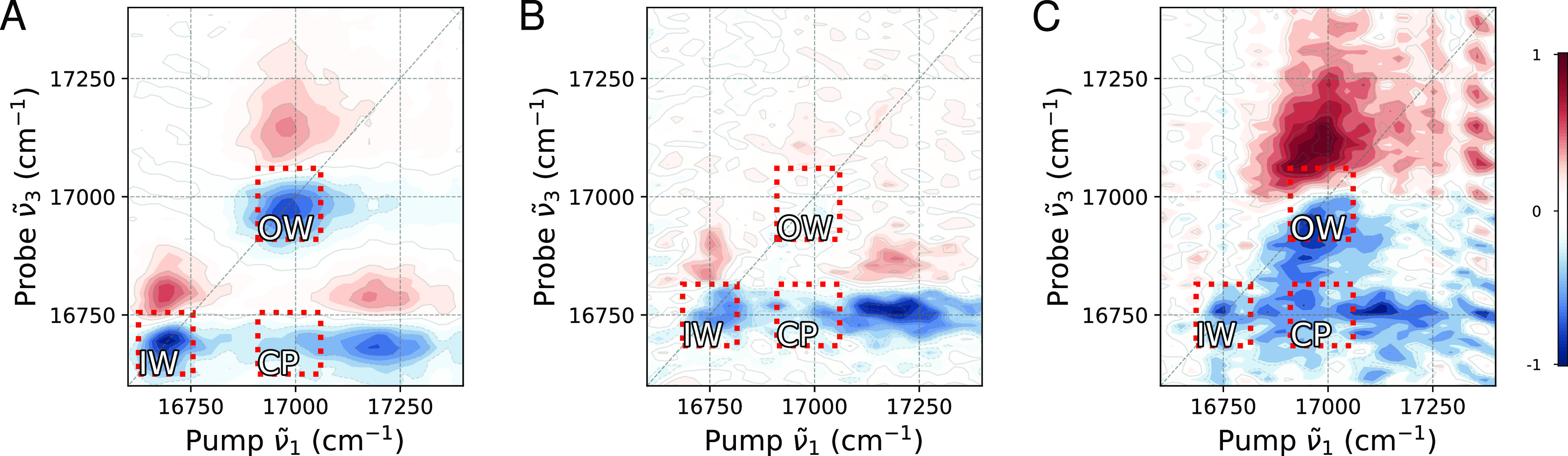
Polarization-resolved 2D spectroscopy of recovering OWs at three stages of the reassembly process, where panel (A) corresponds to DWNTs, while panels (B, C) correspond to isolated IWs (∼40 s after flash-dilution), and recovering nanotubes measured 3 min after flash-dilution, respectively. Ground-state bleach, stimulated emission (GSB, SE), and excited-state absorption (ESA) signals are depicted with negative (blue color) and positive (red color) amplitudes, respectively, to signify the associated change of optical density due to the interaction with the pump pulse. The pump and probe pulses have polarizations orthogonal and parallel to the flow direction in the microfluidic channel, respectively. The spectra shown correspond to ∼160 fs waiting time between the pump and the probe and are normalized to the absolute minimum amplitudes. The signal amplitude is depicted on a color scale ranging from −1 to 1, with increments of 0.1 shown by contour lines. The red dashed lines highlight the IW diagonal, OW (patches in panel (C)) diagonal, and cross-peak (CP) regions. 2D spectra at other waiting times and polarizations of the pump and probe pulses are shown in Figure S12.

2D vis spectra on reforming NTs should be able to confirm the direct attachment of dissolved OW molecules on the IW by the detection of energy transfer between the two. This would manifest as a cross-peak similar to previously observed energy transfer from the OW to the IW of intact DWNTs.^26,28^ In this regard, we performed 2D spectroscopy at ∼40 s (Figure 4B) and ∼3 min (Figure 4C) after flash-dilution (see also Section 4.7). The 2D spectrum of DWNTs (Figure 4A) before flash-dilution is also presented for reference. Here, the alignment of the recovering NTs in the microfluidic channel controls the amplitudes of diagonal and cross-peaks with the use of linearly polarized pump and probe pulses (with respect to the flow direction).

For complete nanotubes, the lowest excitonic transitions (i.e., at the bottom of the exciton band belonging to the IW and OW) are known to be polarized parallel to the NT’s long axis. In contrast, disordered structures, such as patches, have not yet developed any preferential alignment of their transient dipole moment. As a result, both parallel and orthogonally polarized pump excites patches similarly. However, excitation of the intact IW is minimized by setting the pump polarization orthogonal with respect to the flow direction of the sample. This makes the orthogonal pump polarization preferential for isolating the contribution of the disordered species. As the energy transfer is expected to occur from the patches to the IW, the probe polarization should be set parallel to highlight the cross-peak at the frequency of the IW response.

For DWNTs (Figure 4A), the diagonal peaks (*ν̃*_1_ = *ν̃*_3_ = 16,700 cm^–1^ and *ν̃*_1_ = *ν̃*_3_ = 17,000 cm^–1^) are the lowest exciton transitions in the IW and OW, respectively,^10,45^ which are polarized along the NT long axis^10,25^ and, due to imperfect alignment of the NT with the flow direction, are weakly excited by the orthogonal polarized pump pulse.^25,35^ The cross-peak (*ν̃*_1_ = 17,000 cm^–1^, *ν̃*_3_ = 16,700 cm^–1^) indicates population transfer from the OW to the IW. There is also a noticeable cross-peak at *ν̃*_1_ = 17,200 cm^–1^ and *ν̃*_3_ = 16,700 cm^–1^, associated with ultrafast (<100 fs) relaxation of the higher-lying excitonic transitions onto the IW,^45^ where these higher-lying transitions are (partially) polarized perpendicular to the NT long axis,^10,25^ and hence are strongly excited by the orthogonal polarized pump pulse.

After microfluidic flash-dilution^3,22^ (Figure 4B), the OW diagonal peaks (*ν̃*_1_ = *ν̃*_3_ = 17,000 cm^–1^), as well as the cross-peak (*ν̃*_1_ = 17,000 cm^–1^, *ν̃*_3_ = 16,700 cm^–1^), are hardly observed as expected, while only the IW peaks are retained, which undergo a ∼60 cm^–1^ blue shift.^3,22^ The OW diagonal peak (*ν̃*_1_ = *ν̃*_3_ = 17,000 cm^–1^) and the corresponding cross-peak (*ν̃*_1_ = 17,000 cm^–1^, *ν̃*_3_ = 16,700 cm^–1^) begin to recover ∼3 min after flash-dilution (Figure 4C). The very presence of the cross-peak corroborates the attachment of the molecular patches on the IW as otherwise energy transfer from the former to the latter would not have been possible. Furthermore, the broad elliptical shape of the diagonal OW peak (Section S11) reveals that there is more inhomogeneous disorder in recovering OWs than in the initial OWs. This observation is compatible with the formation of patches, as each patch will feel a different local environment.

## 3 Conclusions

In this paper, we used a unique combination of experimental and theoretical approaches to study out-of-equilibrium self-assembly processes. We focused on the C8S3 DWNT system, whose structural and optical properties are well-known, and studied the reassembly of the outer wall after having removed it by flash-dilution. We observed fast (within 1 min of flash-dilution) formation of patches by dissolved outer nanotube molecules arriving on the intact inner nanotube. The molecules within each patch are placed in a well-ordered fashion, while the patches themselves have random orientations with respect to each other. After the outer wall is rebuilt with patches, a slower process (>2 min) of the alignment and merging of patches begins.

A detailed understanding of self-assembly processes presented here plays an important role in unraveling the formation of DWNTs from individual C8S3 molecules. For example, the formation of DWNTs from individual C8S3 molecules most probably begins with disordered intermediate structures similar to the ones presented in this research. The orientation of transition dipole moments, as well as the energy transfer process occurring in such intermediate structures, can be studied in a microfluidic setting using transient linear dichroism and polarized 2D vis spectroscopy methods similar to those presented in this study; such experiments are already underway. The present study also opens up advancing research in the engineering of self-assembly materials for a wide range of applications. For example, architecture design of the nanotubes appears feasible by adding quantum dots or other functional compounds between the walls without restricting self-assembly of the OW. In a broader context, this work constitutes a powerful platform to study out-of-equilibrium self-assembly processes in real time, while providing insights into both the aggregate shape and molecular packing.

## 4 Methods

### 4.1 Materials and Sample Preparation

Double-walled nanotubes were prepared following the alcoholic route.^3^ First, high-concentration stock solutions (*c* = 2.32 × 10^–3^ M) of the dye 3,3′-bis(2-sulfopropyl)-5,5′,6,6-tetrachloro-1,1′-dioctylbenzimidacarbocyanine (C8S3, *M* = 903 g mol^–1^; FEW Chemicals, Germany; used as received) were prepared in methanol (MeOH, Biosolve BV). To induce aggregation into double-walled nanotubes, 130 μL of the stock solution was added to 500 μL of Milli-Q water, gently swirled, and left in the dark for 24 h. Thereafter, another 500 μL of Milli-Q was added to the sample solution. This resulted in a final molar concentration of the sample of *c* = 2.67 × 10^–4^ M and a MeOH content of 9 wt % (12 vol %).

### 4.2 Microfluidic Flash-Dilution and Nanotube Recovery

Microfluidic flash-dilution was performed as previously reported;^26,27^ a schematic of the microfluidic setup is shown in Figure 1A. Nanotube sample solution and diluting agent (mixture of Milli-Q water and MeOH, 1:1 by volume) were supplied by syringe pumps (New Era, model NE-300) and mixed in a commercially available tear-drop micromixer (Micronit, The Netherlands) at flow rates of 250 and 420 μL h^–1^, respectively. Under these conditions, the molar concentration of the sample solution was reduced to *c* ≈ 10^–4^ M, and the MeOH content increased to 31 wt % (36 vol %). The flow rate ratio of the sample solution and the diluting agent were set lower compared to previous reports^26,27^ (250 vs 350 μL h^–1^), which we found to result in a more stable dissolution of the outer layer over time. The microfluidic output defines time zero, which is the beginning of the self-assembly of the OWs. For 2D spectroscopic measurements, the mixed sample solution was relayed to two identical thin-bottom microfluidic flow cells (channel thickness 50 μm, channel width 500 μm). The first flow cell was used for experiments on the isolated IWs ∼ 40 s after mixing. The sample solution was then further relayed to a second flow cell for experiments on the partially recovered nanotubes (corresponding to ∼140 s after mixing). Finally, the sample solution was collected in a glass vial for further recovery.

Flash-dilution was also carried out in a standard 1 mm cuvette, where 210 μL of DWNT solution was added with 105 μL of 1:1 (v/v) water–methanol mixture and was vigorously shaken, and the absorption spectra were recorded using a PerkinElmer 900 UV/vis/NIR spectrometer.

For transient linear-dichroism measurements, 200 μL of the DWNT sample was mixed with 1.6 mL of a flash-dilution mixture (2:1 v/v water–methanol mixture) in a bottle, and LD of the recovering OW is measured in a 0.2 mm flow cell. A higher ratio of water in the dilution mixture allowed higher number of inner NTs to remain unaffected by the flash-dilution process. A white LED light was used as a (isotropic) light source. The parallel and perpendicular polarized light was separated after the sample using a polarizing cube (ThorLabs), which allowed simultaneous monitoring of absorption of parallel and perpendicular (with respect to the flow direction) polarized light in a home-built spectrometer.

### 4.3 Cryogenic Transmission Electron Microscopy

Flash-diluted NTs were frozen rapidly at ∼30 s (Figure 1F) and 4.5 min (Figure 1G) after flash-dilution. The protocol for freezing and imaging was the same as described in detail in ref (22). A FEI Tecnai T20 transmission electron microscope was used for imaging along with a LaB6 cathode working at 200 keV. An UltraScan 4000 UHS CDD camera (Gatan, Pleasanton) operating in a low-dose mode was used to record the cryo-TEM images of the acquired samples. The spatial resolution of the microscope was estimated as 0.5 nm.^22^

### 4.4 Molecular Dynamics Simulations

A flash-dilution experiment was simulated using CG MD, providing detailed microscopic insight into the local packing of molecules and their dynamics. Furthermore, with exciton modeling,^32^ the absorption and linear-dichroism spectra were simulated from the CG-MD simulations, which is described in Section 4.6. All MD simulations were performed with the Martini 3 force field^34,37,46^ and Gromacs.2018.1 or Gromacs 2020 package.^47,48^ The initial coordinates for the C8S3 nanotubes were prepared by creating 2d lattices with the C8S3 CG model and rolling them into cylinders.^31^ Each system was solvated in Martini 3 water beads (W, representing four H_2_O molecules), and Na+ (TQ5 bead with a +1 charge) was used to neutralize the charge of each system. Methanol was also modeled with standard Martini 3 parameters (SP2r, representing 2 MeOH molecules). In all systems, the Berendsen barostat^49^ was used to maintain the pressure constant at 1 bar with a time constant of 1 ps and compressibility of 3 × 10^–4^ bar^–1^. The temperature was kept constant at 300 K by using the V-rescale algorithm with a time constant of 0.1 ps.^50^ van der Waals and electrostatic interactions were calculated using the Verlet list scheme^51^ with the cutoff set at 1.1 nm and a buffer tolerance of 0.01 kJ mol^–1^ ps^–1^. Long-range interactions were treated using the reaction field method^52^ with Martini 3 settings epsilon_r = 15 and epsilon_rf = 0, and the bond lengths were constrained by using the LINCS method.^53^ The systems were translated and rotated to remove the center of mass motion of the IW every 100 steps.

A short (20 nm) nanotube was solvated in water–methanol solution (∼10% MeOH/H_2_O mole fraction). Each system was optimized for 1000 steps by using the Steepest Descent algorithm. Then, a short equilibration step followed in the NPT ensemble for 10 ns with a time step of 10 fs. During the equilibration step, restraints (100 kJ mol^–1^) acted on the aromatic core to allow the tails and solvent to relax. Each system was simulated for 1 μs simulation with 20 fs time step to act as reference systems before the OW was removed. After removal of the OW, position restraints were constantly acting on the aromatic core of the IW to maintain its initial configuration. The IW gradually deformed when restraints were not present. The recovery process was mimicked in a series of titration steps by adding a small number (20 or 40) of C8S3 molecules every 0.5–1 μs of simulation (see Table S2 for more details). The new molecules were added by first enlarging the simulation box, adding the new molecules at random positions available in the larger box, and adding neutralizing ions. Before starting production, an energy minimization and short equilibration run (1 ns) with a 5 fs second time step were performed. Here, we report a number of independent titration simulations from which we have obtained reasonable statistics. An overview of the performed simulations is given in Section S6.

### 4.5 Patch Identification

For each frame of an MD trajectory, we identified patches based on the position and orientation of molecules. A patch is defined as a spatial cluster of molecules that are in close proximity and have a similar orientation. To enable direct comparison of positions and orientations, the positions were regularized by the largest dimension of the MD simulation box, and dipole moments were divided by their magnitude such that all input values are dimensionless and lie between −1 and +1. Patches where then found using the density-based spatial clustering of applications with noise (DBSCAN) algorithm.^54,55^ In the DBSCAN algorithm, data points (molecule positions and orientations) are considered to be part of the same cluster (patch) when at least two points lie within a hyperdimensional sphere with a radius of 0.2 (dimensionless). Supplying the algorithm with both spatial and orientation data allowed for the identification of patches that may be spatially separated or connected, while having different molecular orientations, as shown in Figure 3.

The DBSCAN algorithm performed best in a visual comparison with other clustering algorithms that were tested, K-means, mean-shift, Ward hierarchical clustering, OPTICS, and Gaussian mixtures.

### 4.6 Exciton Modeling

The absorption spectra and linear-dichroism spectra were calculated from MD trajectories using an exciton model.^32^ Each C8S3 molecule in the MD trajectory was abstracted to a two-level system with a transition dipole moment between the two levels. The transition dipole moment was placed at the C3 bead and lies in the direction between the NNA and NNB beads of the CG-MD model,^34^ as indicated in Figure 1H by the red arrow. The aggregation of C8S3 molecules is invariant under the inversion of the chromophore core. Hence, we may define the direction of the transition dipole as going from NNA to NNB or vice versa. We chose the transition dipole with the positive direction with respect to the tubular axis (long/parallel axis). The Hamiltonian is then given by
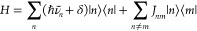
1where *ν̃*_*n*_ is the transition frequency, δ = 350 cm^–1^ is the gas-to-crystal shift, and *J*_*nm*_ is the coupling of transition moments between two molecules. The transition frequency *ν̃*_*n*_ was sampled from a Gaussian distribution centered around the monomer frequency (ℏ*ν̃*_0_ = 19,498 cm^–1^) with standard deviation (σ = 231 cm^–1^), which was parametrized by ref (32). The resonant excitation energy transfer coupling *J*_*nm*_ was determined by using extended dipole moments. Each transition dipole moment μ⃗_*n*_ was deconstructed into positive and negative charges (*q* = 0.34 *e*) separated by a distance *l* = 7 Å in the direction of the transition dipole moment (such that |μ⃗|= *ql* = 11.4 D).^25^ This leads to the following expression for the coupling
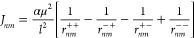
2where *r*_*nm*_^±±^ is the distance between the different charges and α = 5.04 × 10^–3^ cm^–1^ Å^3^ D^–2^ is a constant to convert the units to wavenumbers.

The absorption spectrum in a given polarization direction was determined as follows
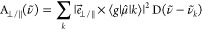
3where *ν̃*_*k*_ is the frequency of eigenstate *k* of the Hamiltonian, *e⃗*_⊥/∥_ is the polarization vector of light, and ⟨*g*|μ̂|*k*⟩ is the transition dipole moment between the ground state *g* and eigenstate *k*. The transition dipole operator μ̂ is defined as ∑_*n*_μ⃗_*n*_(|*n*⟩⟨*g*| + |*g*⟩⟨*n*|) × *D*(*ν̃*) denoting the line shape function that is taken to be a zero-mean Gaussian with standard deviation σ = 75 cm^–1^. The absorption spectrum is averaged over 1000 realizations of the transition energies *ν̃*.

### 4.7 Polarization-Resolved 2D Spectroscopy

Polarization-resolved 2D spectra were recorded on a pulse-shaper-based setup; its design is similar to ref (56). The pump and probe pulses (central wavelength 17,000 cm^–1^, spectral width ∼1,500 cm^–1^) were generated using noncollinear optical parametric amplifiers (NOPAs) seeded by the output of a Ti:sapphire regenerative amplifier (Elite Duo, Coherent) with a pulse repetition rate of 1 kHz. To retrieve the excitation axis, the coherence time was scanned between 0 and 400.4 fs in steps of 0.7 fs. A two-step phase-cycling scheme was adopted to extract the desired third-order signal from all other unwanted signals by setting the changing phase difference between the two pump pulses (Δϕ_12_) between 0 and π for every other pump pulse pair. At a given waiting time, 50 individual spectra were averaged to obtain one 2D spectrum. The pulse energies of the pump and probe pulses were set to Δ*E* = 0.6 and 0.3 nJ, respectively. All experiments were carried out at room temperature. A detailed description of the 2D spectroscopy setup can be found in ref (56).

## Data Availability

All data generated or processed during this study are available from the corresponding author upon reasonable request.

## Supporting Information Available

Absorption spectra after flash-dilution, mixed-wall line profiles in TEM images, modeling of transient linear dichroism, rate equations for OD, LD, and LDr signals, LDr of the isolated inner wall, overview of MD simulations, coarse-grained MD model and overview of interactions, relation between CG model simulations and experimental time scales, simulated absorption spectra, polarization-resolved 2D spectroscopy, ellipticity analysis, and references, including Figures S1–S14 and Tables S1–S4 (PDF)
Animation of a CG-MD titration simulation shows patch formation and patch reorientation (AVI)

## Supplementary Material

ja3c07103_si_001.pdf

ja3c07103_si_002.avi

## Footnotes

1 3,3′-Bis(2-sulfopropyl)-5,5′,6,6′-tetrachloro-1,1′-dioctylbenzimidacarbocyanine.
