# Supplementary material for: Watching Molecular Nanotubes Self-Assemble in Real Time
Source: J Am Chem Soc. 2023 Oct 6;145(41):22494–503. doi: 10.1021/jacs.3c07103 (PMC10591479; doi:10.1021/jacs.3c07103)
Supplement: Supplementary file 1 — ja3c07103_si_001.pdf [file ja3c07103_si_001.pdf]

Supplementary Material

Watching Molecular Nanotubes Self-Assemble in Real Time

Marìck Manrho,[1] Sundar Raj Krishnaswamy,[1] Björn Kriete,[1] Ilias Patmanidis,[2,3] Alex H. de Vries,[2] Siewert J. Marrink,[2] Thomas L. C. Jansen,[1] Jasper Knoester,[1,4] and Maxim S. Pshenichnikov[1]

[1)]*University of Groningen, Zernike Institute for Advanced Materials, Nijenborgh 4, 9747 AG Groningen, The Netherlands*

[2)]*University of Groningen, Groningen Biomolecular Sciences and Biothechnology Institute, Nijenborgh 7, 9747 AG Groningen, The Netherlands*

[3)]*Aarhus University, Department of Chemistry, Langelandsgade 140, 8000 Aarhus C, Denmark*

[4)]*Leiden University, Faculty of Science, Einsteinweg 55, 2300 RA Leiden, The Netherlands*

(*Electronic mail: m.s.pchenitchnikov@rug.nl.)

(Dated: 4 October 2023)

CONTENTS

S1. Absorption Spectra After Flash Dilution 3

S2. Mixed Wall Line Profiles in TEM Images 4

S3. Modelling of Transient Linear Dichroism 5

S4. Rate equations for OD, LD, and LD_r signals 5

S5. LD_r of Isolated Inner Wall 8

S6. Overview of MD simulations 10

S7. Coarse grained MD model and overview of interactions 11

S8. On the relation between the CG model simulations and experimental time scales 12

Stage A: Diffusion and formation of C8S3 oligomers or aggregates in solution. 12

Stage B: Diffusion of C8S3 molecules on the IW and formation of small ordered patches. 17

S9. Simulated Absorption Spectra 19

S10. Polarization-Resolved 2D Spectroscopy 22

S11. Ellipticity analysis 24

References 26

S1. ABSORPTION SPECTRA AFTER FLASH DILUTION

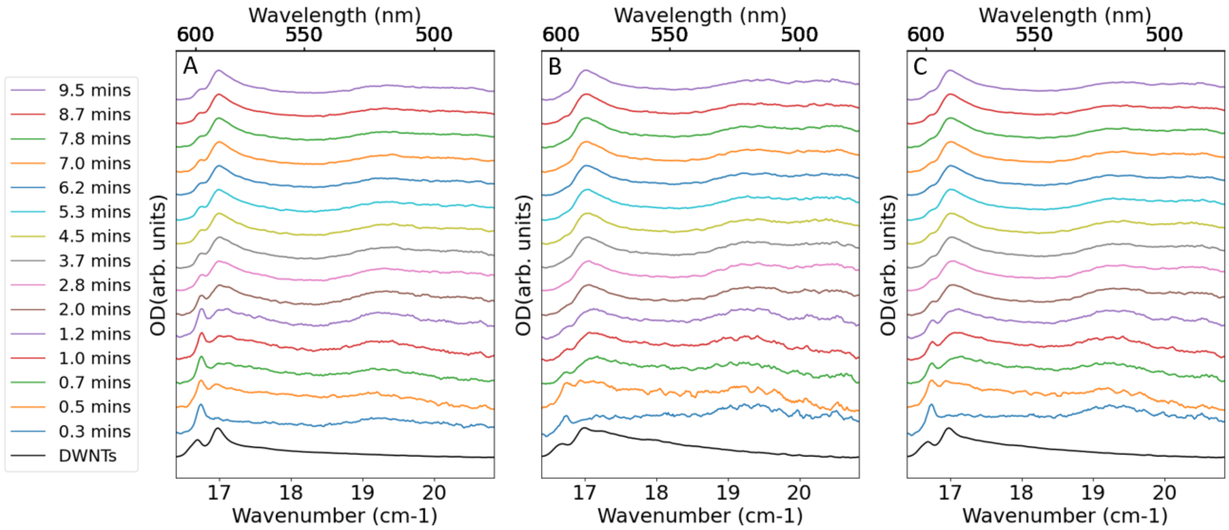

FIG. S1. Parallelly polarised (A), perpendicularly polarised (B) and isotropic (C) absorption spectra at several times after flash dilution. The color of the line represents the time after flash dilution in minutes. The black line is the absorption spectrum of the DWNTs before flash-dilution. Each plot is normalised by the maximum optical density and is offset for better visualisation.

Fig. S1 shows polarised and isotropic absorption spectra normalised to maximum optical density at different times after flash-dilution, where 200 μL of DWNT solution was mixed with 1.6 mL of 2:1 (v/v) water-methanol mixture. The resulting concentration of methanol in the final solution was $\sim$ 40 vol.%. The recovering outer wall (OW) is observed spectroscopically by the growing OW absorption peak at $\sim$ 17000 cm^{-1}. Allowing even more time for the NTs to recover, the original DWNT spectrum is eventually restored, but with a lower amplitude (not shown in SI Fig. S1). The latter is a direct consequence of the increased MeOH content of the sample solution due to flash-dilution, which shifts the equilibrium point between monomers and NTs, in favour of the former. The monomer peak does not decrease monotonically throughout the experiment, which can be explained by local fluctuations in MeOH concentration during flash-dilution. The changes in optical density, linear dichroism and reduced linear dichroism of the recovering OW at different times after the above flash-dilution experiment are discussed in Section 2.3 in the main text.

S2. MIXED WALL LINE PROFILES IN TEM IMAGES

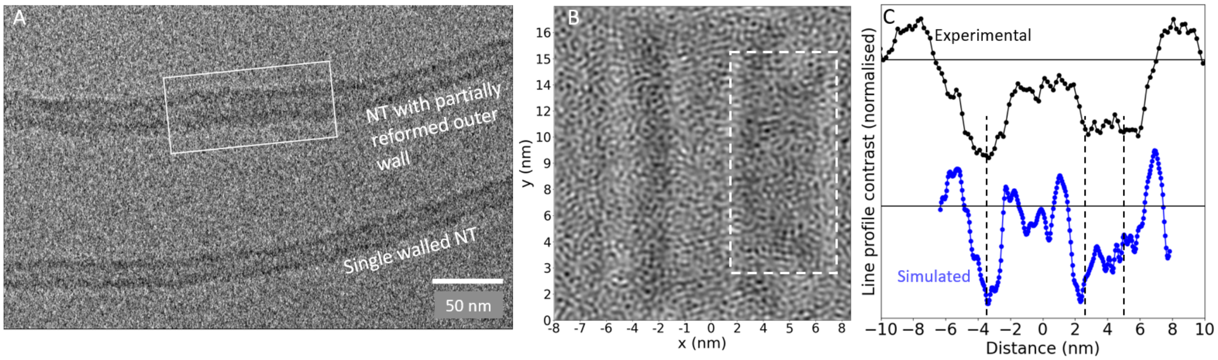

FIG. S2. Experimental cryo-TEM image (A) and simulated high resolution TEM (HRTEM) image (B) of NTs along with their respective line profiles (C). The experimental cryo-TEM image (A) of C8S3 nanotubes is taken approximately two minutes after flash-dilution, where the image has both single-walled NT and NT with partial OW reformation. A section from the latter (highlighted by solid white rectangle, A) is considered and the line profile of the same is shown in C. The line profile of simulated HRTEM image of a nanotube (B) with partial outer nanotube reformation (highlighted by dashed white rectangle, B) is shown in C (blue). Line profiles (C) are normalised to a minimum of -1, and are offset for the ease of comparison. The black dashed lines indicate the minima of the experimental line profiles.

Fig. S2A shows the experimental cryo-TEM image of C8S3 nanotubes approximately two minutes after flash dilution. The image consists of a single-walled NT, as well a NT with partial reformation of the OW. A section (highlighted by solid white rectangle, Fig. S2A) is considered from the latter for line profile analysis, and is shown in Fig. S2C. The procedure to obtain the line profiles is detailed in Ref. S1. Fig. S2B shows simulated high-resolution TEM (HRTEM) image of a nanotube with partial OW reformation (highlighted by dashed white rectangle), obtained using recently developed abTEM program[S2], while Fig. S2C shows the line profile of the same. The details about the simulated HRTEM image can be found in ref. S1. The experimental and simulated line profiles bear close resemblance to each other, while the minima of line profiles (dashed black lines) are also within proximity (< 0.5 nm) to each other. These observations confirm that the experimental cryo-TEM image indeed corresponds to a nanotube with partial OW reformation.

S3. MODELLING OF TRANSIENT LINEAR DICHROISM

In this section we give the definition of the quantities shown in Fig. 2, namely optical density (OD), linear dichroism (LD), and reduced linear dichroism (LD_r). The optical density is given by the isotropic absorption averaged over the $\tilde{\nu}$ range $[\tilde{\nu}_a, \tilde{\nu}_b]$ and is given by

$$\langle \mathrm{OD}(t) \rangle = \frac{\int_{\tilde{\nu}_a}^{\tilde{\nu}_b} \left(\mathrm{A}_{\parallel}(\tilde{\nu}, t) + 2\mathrm{A}_{\perp}(\tilde{\nu}, t) \right) d\tilde{\nu}}{\tilde{\nu}_b - \tilde{\nu}_a}, \tag{S1}$$

where $\mathrm{A}_{\parallel}$ and $\mathrm{A}_{\perp}$ are the polarised absorption spectra in the parallel or perpendicular direction with respect to the fluid flow in the microfluidic flow cell (see Fig. 1A), respectively. The LD is given by the difference in absorption in the parallel and perpendicular directions,

$$\langle \mathrm{LD}(t) \rangle = \frac{\int_{\tilde{\nu}_a}^{\tilde{\nu}_b} \left(\mathrm{A}_{\parallel}(\tilde{\nu}, t) - \mathrm{A}_{\perp}(\tilde{\nu}, t) \right) \mathrm{d}\tilde{\nu}}{\tilde{\nu}_b - \tilde{\nu}_a}. \tag{S2}$$

The reduced linear dichroism is the ratio between LD and OD,

$$\langle \mathrm{LD}_r(t) \rangle = \frac{\langle \mathrm{LD}(t) \rangle}{\langle \mathrm{OD}(t) \rangle}. \tag{S3}$$

In the next section of this SI we provide models for these quantities.

S4. RATE EQUATIONS FOR OD, LD, AND LD_r SIGNALS

In this subsection, we present the rate equations underlying the model fits shown in Fig. 2 by the dotted green and dashed red lines. First, we will model the optical density for the recovering OW and newly created DWNTs. Second, the LD response is modelled with and without an intermediate disordered state.

The isotropic absorption can be separated into three contributions, namely the inner wall (IW) which remains after flash dilution, the recovering OW (ROW) which reforms around the IW and newly formed DWNTs growing from monomers. This is written as follows,

$$\langle \mathrm{A}(t) \rangle = \left\langle \mathrm{A}^{\mathrm{IW}} \right\rangle + \left\langle \mathrm{A}^{\mathrm{ROW}}(t) \right\rangle + \left\langle \mathrm{A}^{\mathrm{DWNT}}(t) \right\rangle, \tag{S4}$$

where the angled brackets denote the average over wavenumbers. We assume that the response of the ROW and newly formed DWNTs are constant over this wavenumber range and only depend on time. This means that the red shift in absorption (J-aggregate) must converge very quickly as

a function of OW growth. The IW is barely affected by the flash dilution. Hence, we take the contribution of the IW to be constant in both wavenumber and time.

We assume that new DWNTs are formed from monomers in solution at a constant rate γ. The number of newly formed DWNTs is then given by,

$$\mathrm{N}^{\mathrm{DWNT}}(t) = \gamma t. \tag{S5}$$

In practice the growth of new DWNTs is limited by the number of monomers which should yield exponential growth. However, we assume the growth of new DWNTs remains in the linear regime.

The ROW grows in a different way as its growth is restricted by the remaining IW. It is assumed that the probability for a monomer to adsorb to the ROW is proportional to the number of available locations for that molecule to occupy and that the concentration of monomers is constant. The fraction of ROW is then given by

$$\mathrm{N}^{\mathrm{ROW}}(t) = 1 - e^{-\alpha t}, \tag{S6}$$

where α is the rate of monomer adsorption.

Assuming all molecules in the OWs contribute equally to the optical density within the wavelength range of interest we can write

$$\left\langle \mathrm{A}^{\mathrm{DWNT}}(t) \right\rangle = \mu^2 \mathrm{N}^{\mathrm{DWNT}}(t) \tag{S7a}$$

$$\left\langle \mathrm{A}^{\mathrm{ROW}}(t) \right\rangle = \mu^2 \mathrm{N}^{\mathrm{ROW}}(t), \tag{S7b}$$

where μ is the average absorption cross section of a complete OW within the wavenumber range of interest. The result of Eqs. S7 are shown individually in Fig. S3A.

Now we turn our attention to a model for the linear dichroism. The linear dichroism signal is computed similarly to the absorption signal, namely

$$\langle \mathrm{LD}(t) \rangle = \left\langle \mathrm{LD}^{\mathrm{IW}} \right\rangle + \left\langle \mathrm{LD}^{\mathrm{ROW}}(t) \right\rangle + \left\langle \mathrm{LD}^{\mathrm{DWNT}}(t) \right\rangle. \tag{S8}$$

We first construct a model for the linear dichroism when the OW reassembles with ordered transition dipole moments. In this case the LD signal is simply the isotropic absorption scaled by some factor ρ,

$$\left\langle \mathrm{LD}^{\mathrm{ordered}}(t) \right\rangle = \rho \left\langle \mathrm{A}(t) \right\rangle. \tag{S9}$$

For the second case we assume an intermediate disordered state. In this disordered state only $\mathrm{N}^{\mathrm{ordered}}$ molecules are ordered, and thus contribute to the LD signal, while all other molecules are

randomly oriented and therefore do not contribute to the LD signal. The rate at which the ordered part grows is proportional to the number of disordered molecules present in the OW. This leads to the following differential equation for the percentage of ordered molecules $\mathrm{N}^{\mathrm{ordered}}$,

$$\frac{\partial \mathrm{N}^{\mathrm{ordered}}(t)}{\partial t} = \beta \left(\mathrm{N}^{\mathrm{OW}}(t) - \mathrm{N}^{\mathrm{ordered}}(t) \right), \tag{S10}$$

where β is the rate at which a disordered molecule becomes ordered. The above equation is solved for $\mathrm{N}^{\mathrm{DWNT}}$ and $\mathrm{N}^{\mathrm{ROW}}$ separately. Inserting Eqs. S5 and S6 into Eq. S10 we find

$$\mathrm{N}^{\mathrm{ordered}}_{\mathrm{DWNT}}(t) = \frac{\gamma}{\beta} \left(e^{-\beta t} - 1 \right) + \gamma t \tag{S11a}$$

$$\mathrm{N}^{\mathrm{ordered}}_{\mathrm{ROW}}(t) = \frac{\beta e^{-\alpha t} - \alpha e^{-\beta t}}{\alpha - \beta} + 1. \tag{S11b}$$

The LD signals from the newly formed DWNTs and recovering OW are then simply

$$\left\langle \mathrm{LD}^{\mathrm{DWNT}}(t) \right\rangle = \rho \mu^2 \mathrm{N}^{\mathrm{ordered}}_{\mathrm{DWNT}}(t) \tag{S12a}$$

$$\left\langle \mathrm{LD}^{\mathrm{ROW}}(t) \right\rangle = \rho \mu^2 \mathrm{N}^{\mathrm{ordered}}_{\mathrm{ROW}}(t). \tag{S12b}$$

The result of Eqs. S12 is shown in Fig. S3B.

For the experimental fit, the average absorption and LD of the IW are obtained by the experimental value at time zero. All other parameters are free fit parameters where parameters α and γ have to fit the OD and LD simultaneously.

Figure S3 shows the individual components of Fig. 2A-B in the main text. Figure S3B follows logically from the modelling of Fig. S3A when assuming a disordered intermediate state, as is shown in the paragraphs leading up to Eqs. S12. In Eq. S12a it is assumed that even in newly formed DWNTs the molecules need some time to reorient into an ordered configuration. The modelling however does not provide clear evidence that this is the case as the LD of the newly formed DWNTs still grows roughly linear in the time frame considered here.

In Fig. S4 we present the same data as Fig. 2D-F but as a function of simulation time instead of the number of molecules of the OW. The trend of the data as a function of time is similar to the trend as a function of N_{OW} for OD, LD and LD_{r}. The average absorption as a function of time shows the variations in the rate at which new molecules are added to the simulation box (see also SI section S6).

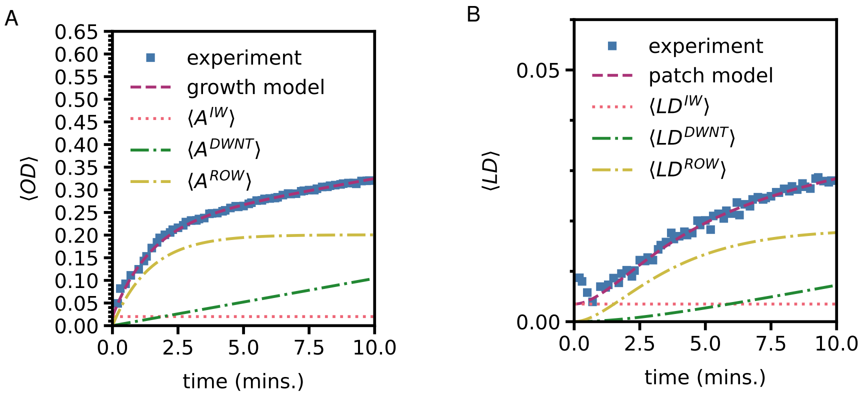

FIG. S3. Decomposition of A) absorption and B) linear dichroism models. There are three contributions to the models, namely the constant IW (red dotted), newly formed DWNT (dashed green), and the recovering OW (dashed yellow) from Eqs. S11 and S12. The parameters used are shown in Table S1.

Parameter	Experimental fit value	Multi-scale modelling fit value
α	0.700 min^{-1}	9.5×10^{-3} N^{-1}
β	0.400 min^{-1}	3×10^{-3} N^{-1}
γ	0.052 min^{-1}	0 N^{-1}
μ	0.448	11.4 D
$\langle \mathrm{A}^{\mathrm{IW}} \rangle$	0.020	0.442
$\langle \mathrm{LD}^{\mathrm{IW}} \rangle$	0.0035	0.110
ρ	0.092	0.5

TABLE S1. Parameters for the rate equation models for the experimental and MD simulation fits shown in Fig. 2 and Fig. S3.

S5. $\mathrm{LD_r}$ OF ISOLATED INNER WALL

The OW self-assembles through a disordered intermediate state as is indicated in Fig. 2 in the main text. Here, similar analysis is presented (as discussed in Section 2.3) for the spectral region of the IW peak (16600 cm^{-1}-16750 cm^{-1}) as is shown in Fig. S5. Formation of new DWNTs results in an increase in the OD (Fig. S5A) as well as LD (Fig. S5B), while the overlapping contribution from the reforming OW (16892 – 17094 cm^{-1}) contributes only to an increase in the optical density and not linear dichroism in the initial time after flash-dilution (< 2 minutes), as

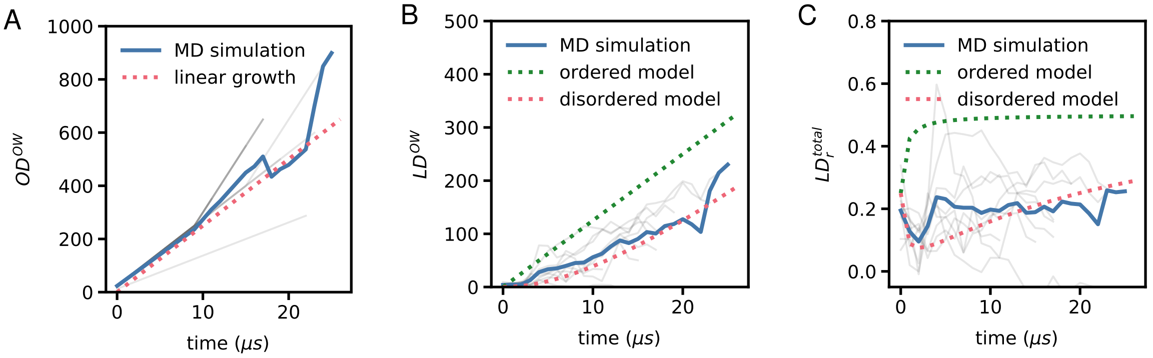

FIG. S4. Isotropic absorption (A), linear dichroism (B) and reduced linear dichroism (C) from CG MD simulations as function of time. The grey lines show the signals from the different simulations. The blue line is the average of the various simulations. The dotted lines represent model fits.

the OW reformation occurs in a disordered fashion in the beginning, where the transition dipole moment of the attached molecules are randomly oriented. Therefore, LD increases at a slower rate than OD. As a consequence, the resulting LD_r for the IW peak (Fig. S5C) monotonically decreases, unlike the OW peak shown in Fig. 2C where the LD_r first decreases and then continues to increase over time. The above findings clearly indicates that the IW structure remains roughly constant over the course of the flash-dilution experiment.

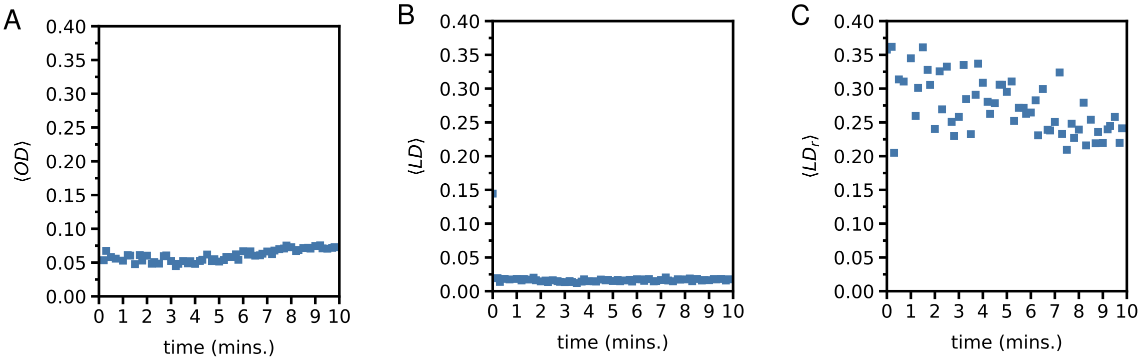

FIG. S5. Optical density (OD), linear dichroism (LD), and reduced linear dichroism (LD_r) averaged over the region of the IW peak (16600 cm^{-1}-16750 cm^{-1}). The structure of the IW is largely unaffected after the flash-dilution process, and the LD_r values monotonically decrease due to the overlapping contribution from the reforming (disordered) OW.

S6. OVERVIEW OF MD SIMULATIONS

In table S2 we give an overview of the hyperparameters for each of the MD simulations used for exciton modelling.

Simulation	Addition rate μs^{-1}	Length (μs)	Timestep (μs)	Final N^{OW}
1	10	24	1	240
2	20	25	1	500
3	20	21	1	420
4	Up to 16 μs: 20<br>After 16 μs: 40	27	1	720
5	Up to 10 μs: 20<br>After 10 μs: 40	16	1	440
6-8	Up to 10 μs: 20<br>After 10 μs: 40	18	1	520
9	Up to 10 μs: 20<br>After 10 μs: 40	13	1	280
10-14	40	15	0.5	300
15-19	80	7	0.5	280

TABLE S2. Hyperparameters for each MD trajectory. The addition rate gives the number of molecules added per μs, the length is the total time the simulation ran for, the timestep is the time between adding new batches of molecules, and the final N^{OW} is the number of OW molecules at the end of the simulation.

S7. COARSE GRAINED MD MODEL AND OVERVIEW OF INTERACTIONS

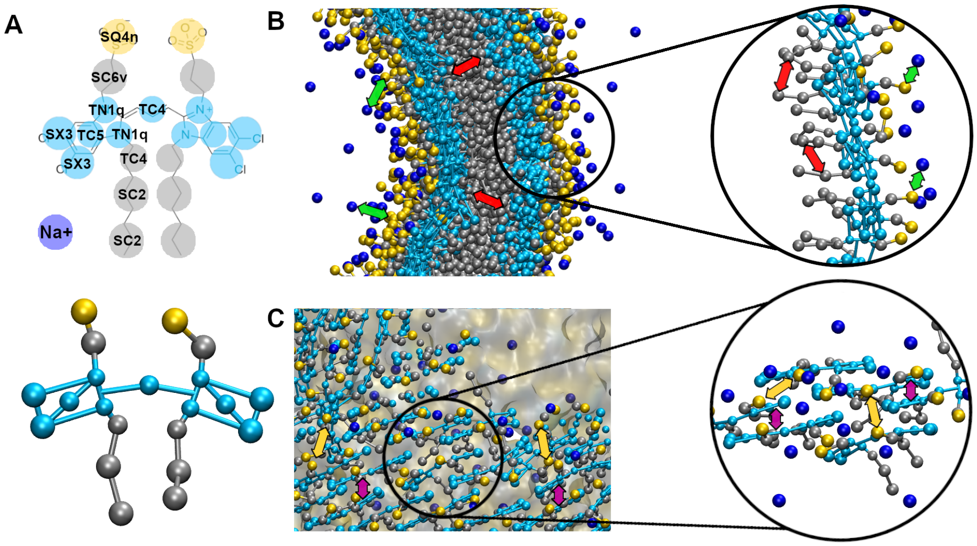

FIG. S6. Overview of C8S3 Martini model and interactions in the system. (A) Coarse grained C8S3 Martini model. Top: specification of Martini model bead types and mapping scheme shown on an atomistic representation of the molecule. For more details, see Ref.[S3]. Bottom: ball-and-stick model representation as used in panels B and C. The main classification of interactions of this molecule are electrostatic from the charged sulfonate groups (yellow) and sodium counter ions (dark blue), electrostatic and steric stacking from the aromatic core (light blue), and dispersion interactions from the hydrocarbon tail (gray). (B) View of a section of the tube along the tube axis, highlighting the alkyl-alkyl tail interaction between the forming OW and the IW indicated by red block arrows. This interaction is expected to be the dominant interlayer interaction. Other interactions are ion-ion interactions between $SO3^-$ and Na^+ ions indicated by green block arrows. The inset shows the interactions once more, also highlighting lateral alkyl-alkyl interactions within one layer. (C) View looking down on the partially reformed outer wall. The inner wall is represented by a smoothened surface. Intermolecular interactions between molecules forming the OW include ion-ion interactions between sulfonate head groups (yellow block arrows) and charged aromatic cores (not shown), aromatic stacking interactions (purple block arrow), and lateral alkyl-tail interactions (not shown, but shown in the inset of panel B); further interactions are electrostatic interactions between sulfonate head groups and Na^+ counterions, as well as polar interactions with solvent (not shown). The interactions are such that the molecules prefer to adopt a brickwork packed structure; however, domain boundaries exist and disorder is visible on the edges of a patch.

S8. ON THE RELATION BETWEEN THE CG MODEL SIMULATIONS AND EXPERIMENTAL TIME SCALES

Here, we used the Martini coarse-grained (CG) model to get molecular insight in the self-assembly process that forms the OW on an intact IW. We performed several types of simulations with the aim to link up different stages in the reassembly process and to attempt to estimate time scales for different stages. It should be clear from the outset that the experimental time scale upon which the reduced linear dichroism (LD_r) signal recovers, being minutes, as well as the experimental length scale of the tubes, being multiple micrometers and multiple tubes, is well out of reach of our CG model. The best we can do at present is to study local (10–100 nm long tube fragments) and relatively short time (our longest titration simulation is 27 μs) events. In this section, we discuss the relation of the CG model to the experiment, emphasizing aspects in which the model may be deficient and to which extent we were able to estimate the effects of the deficiencies on how we interpret the experiment.

Stage A: Diffusion and formation of C8S3 oligomers or aggregates in solution.

After flash dilution, surviving intact IWs of the tube are surrounded by a solution of C8S3 molecules. The first step in the OW reassembly is diffusion of single C8S3 molecules toward the IW surface. Our main C8S3 coarse grain molecular dynamics (CG MD) simulation experiments model arrival of C8S3 molecules on reassembling tubes in bursts, adding 20–40 molecules to the system at a time, and giving them 0.5–1 μs to find their way to the tube, adsorb on the IW surface and join already existing OW patches, explore the IW surface, or form new OW patches. The molecules are added at the edges of the periodic box, while the tube is in the center. Typical box sizes are 20 nm $\times$ 20 nm in the lateral direction; the maximum distance to the tube is then approximately 6.5 nm. New molecules are added at around this distance from the tube.

In most titration steps, all molecules added to the system reached the tube and adsorbed on the tube well within the 0.5–1 μs given. This is consistent with the diffusion coefficient of single C8S3 molecules in the solvent, which is on the order of 10^{-10}–10^{-9} m^2s^{-1}, leading to a diffusion time from the edge of the box to the tube surface on the order of tens of ns in the random walk model.

In some cases, molecules formed small aggregates and remained in solution at the end of the

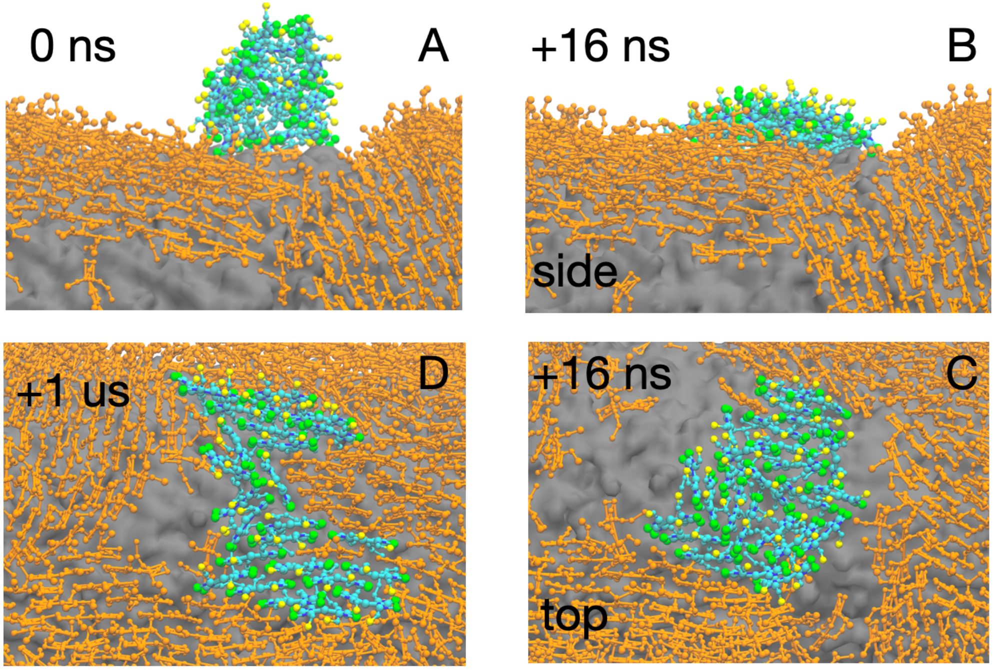

FIG. S7. Quick wetting by small micelle arriving on an empty region. (A) Arrival on the surface. (B, C) Side and top view 16 ns after first touching of the surface. (D) Incorporation of the small micelle in the surface patches 1 μs after arrival. Molecules already on the surface in orange; molecules in the small micelle are colored differently, with the core in blue, sulfonate in yellow and chlorine in green.

titration step. In a subsequent titration step, these small aggregates did reach the tube and adsorbed on it. Once small aggregates arrived on the surface, the time it took for them to fully adsorb on the IW depended on where the aggregate attached. Wetting was almost instantaneous for an aggregate of 26 molecules that attached at an empty region on the IW, and in a further 1 μs, the molecules had joined existing patches. A larger aggregate of 68 molecules that attached on the rim of the tube at a position that already had an OW and whose molecules were ordered with a different rolling angle than the attaching aggregate took considerably longer (approx. 0.5 μs) to wet the IW surface because it needed to displace molecules in the existing, fairly large patch. From visual inspection, the time-limiting step appears to be alignment of the chromophores in the attaching aggregate and existing IW patch. Snapshots of these processes are shown in Figs. S7 and S8.

In terms of realism, there are a number of aspects to take into account:

1. diffusion of single molecule in solution

We tested the diffusion coefficient of a single C8S3 molecule in the solvent, both in the CG and in an all atom (AA)[S3] model at three different temperatures by determining the slope of the mean

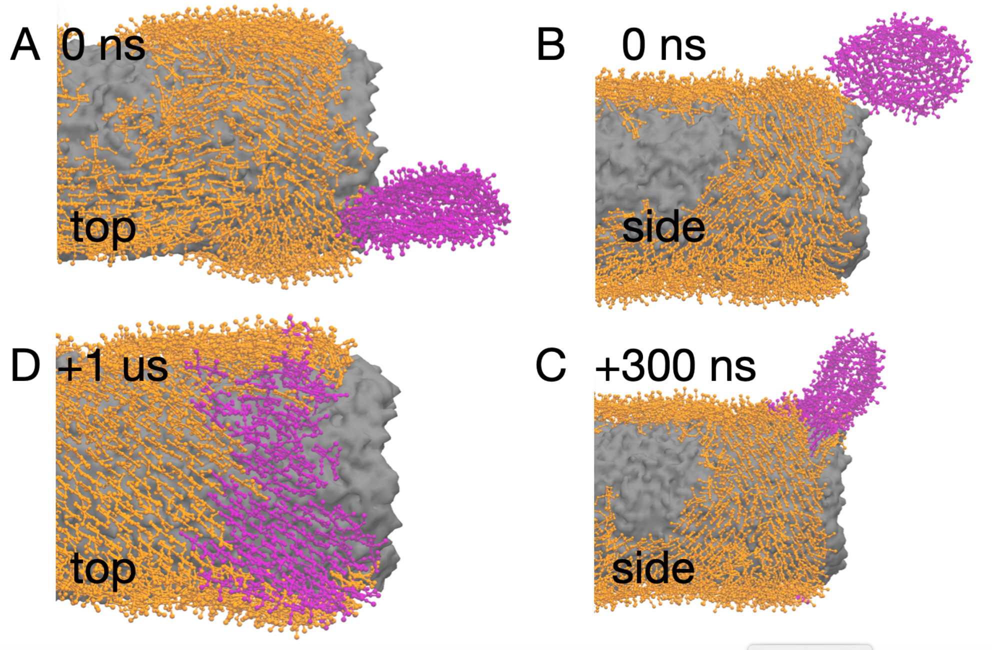

FIG. S8. Slow incorporation of small micelle arriving at an already occupied part of the tube. (A, B) Top and side view of the arrival on the surface. (C) 300 ns after first touching of the surface. (D) Incorporation of the large micelle in the surface patch 1 μs after arrival. Molecules already on the surface in orange; molecules in the small micelle are in purple.

squared displacement (MSD) as a function of time. We found that the diffusion coefficients of single molecules in CG and AA models were on the same order of magnitude at all temperatures. In addition, the diffusion coefficients of the models are of the same order of magnitude as may be expected experimentally for molecules of this size, for example by the Stokes-Einstein model. The diffusion coefficients are reported in Table S3.

Temperature (K)	D $\left(10^{-10}\ \mathrm{m^2 s^{-1}}\right)$ AA	D $\left(10^{-10}\ \mathrm{m^2 s^{-1}}\right)$ CG
300	5 ± 1	5 ± 1
350	13 ± 2	8 ± 1
400	22 ± 6	20 ± 5

TABLE S3. Diffusion coefficients of single C8S3 molecule in aqueous solution in full atomistic detail and in CG model at three different temperatures.

2. concentration and distribution of C8S3 in solution

By adding molecules on the rim of the periodic simulation box (with the tube always in the

middle of the box), the distribution of the C8S3 molecules at the start of each titration step is not uniform and is unlikely to conform to a distribution that occurs near the tube during the re-assembly process. The mixing in microfluidic devices has been the subject of Computational Fluid Dynamics (CFD) simulations, for example[S4], which suggest that the mixing of the stock DWNT solution and diluting MeOH/water mixture will result in a complex non-homogeneous distribution of surviving IW tubes, C8S3 monomers and small aggregates, and provides a perspective on the local space and time character of our approach.

The current and previous experiments and detailed atomistic (AA) MD modeling of double walled nanotubes (DWNTs) provide a number of parameters that can be used to construct a simple random-walk model that provides a reasonable estimate of the arrival of single molecules from solution at the surface of the exposed IW. The first report on the flash dilution experiments[S5] describes the calculation of the concentration of C8S3 molecules in the surviving IW and as monomers in solution by two different methods, and give a range between $\rho_{\mathrm{N_{IW}}} = 1.33–2.55 \cdot 10^{-5}$ M C8S3 molecules present in IW and corresponding $\rho_{\mathrm{N_{mon}}} = 9.78–8.55 \cdot 10^{-5}$ M as monomers in solution (Ref. S5, Supplementary Note 1). The concentrations correspond to $0.80–1.54 \cdot 10^{-5}$ and $5.89–5.14 \cdot 10^{-5}$ molecules per nm^3, respectively. Molecular models of the C8S3 DWNT contain approximately 40 C8S3 molecules per nm tube length in the IW. This means that *on average*, one nm of IW tube length is present in a cross section of about 5 μm^2:

$$\mathrm{A_{IW}} \cdot \mathrm{L_{IW}} = \mathrm{V_{IW}} = \frac{\mathrm{N_{IW}}}{\rho_{\mathrm{N_{IW}}}} \tag{S13}$$

$$\mathrm{A_{IW}} = \frac{40}{1\ \mathrm{nm} \times 0.80 \cdot 10^{-5}\ \mathrm{nm}^{-3}} = 5.00 \cdot 10^{6}\ \mathrm{nm}^2 \tag{S14}$$

In a cylinder geometry, this means that on average every tube is served by monomers that are present within a radius of:

$$\mathrm{A_{IW}} = \pi r_{\mathrm{cyl}}^2 \tag{S15}$$

$$r_{\mathrm{cyl}} = \sqrt{\frac{5.00 \cdot 10^{6}\ \mathrm{nm}^2}{\pi}} = 1.26 \cdot 10^{3}\ \mathrm{nm} \tag{S16}$$

Given the concentration of monomers and assuming their homogeneous distribution over the sample, the volume corresponding to 1 nm IW tube length contains 294 molecules:

$$\mathrm{N_{mon}} = \rho_{\mathrm{N_{mon}}} \mathrm{V_{IW}} = 294 \tag{S17}$$

The question now arises how long it takes on average for the IW surface to be fully covered. Per nm tube length, approximately 50 molecules are required to build a complete OW. Advanced

calculations can be made based on first-passage time distributions[S6,S7], but this is beyond te scope of this paper.

We performed random walk simulations, starting with 294 particles uniformly distributed on lattice points spaced by 1 nm in each Cartesian direction and contained within a circle of radius $1.25 \cdot 10^3$ nm, excluding a circle with a radius of 5 nm, which represents a little more than the IW dimension. In the simulation, for each molecule, in each step, the particle was displaced ± 1 nm in both lateral directions, using reflecting boundary conditions at the outer radius. The reflecting boundary conditions were implemented by letting the particle stay at the position it had before the attempted move. Particles were assumed to be adsorbed or to have arrived at the surface of the IW and therefore be part of the reforming OW if the distance from the center was less than 4 nm, which roughly corresponds to the distance from the center of the IW hydrocarbon region. Using the Einstein-Smoluchowski expression for the diffusion coefficient, each time step corresponds to:

$$\mathrm{D} = \frac{\lambda^2}{2\tau} \tag{S18}$$

$$\tau = \frac{\lambda^2}{2\mathrm{D}} = \frac{1\ \mathrm{nm}^2}{2 \cdot 0.5\ \mathrm{nm}^2\mathrm{ns}^{-1}} = 1\ \mathrm{ns} \tag{S19}$$

for a diffusion coefficient $\mathrm{D} = 5 \cdot 10^{-10}\ \mathrm{m}^2\mathrm{s}^{-1}$.

Figure S9 shows the time it takes for N particles to arrive at the IW in 50 independent random walk simulations, as well as the average over the simulations. The pool of particles present at the start represents the number present in a volume corresponding to a tube length of 1 nm and our interest is in the time it takes for the first 50 molecules to arrive, where the assumption is that these will all end up covering the IW and saturate the OW. It can be seen from Fig. S9 that at the concentration of the experiment, it takes 1.0–2.0 milliseconds for 50 particles to arrive on a 1 nm stretch of tube. In our CG MD titration simulations we use a stretch of 20 nm, corresponding to approximately 1000 molecules to saturate the OW. This means that on average, we should let about 0.5-1.0 molecules arrive every microsecond to match the experiment if a homogeneous distribution of C8S3 monomers is assumed. We performed the titration simulations at rates varying from 20 molecules per microsecond to 80 molecules per microsecond (as such or at the rate of 40 molecules per 0.5 μs). This is considerably faster than in the estimations above. The realism of our obtained patch structures and orientations may, however, still be high if diffusion of adsorbed molecules on the surface is sufficiently slow. We investigated the diffusion on the surface, and results are discussed in the discussion of Stage B.

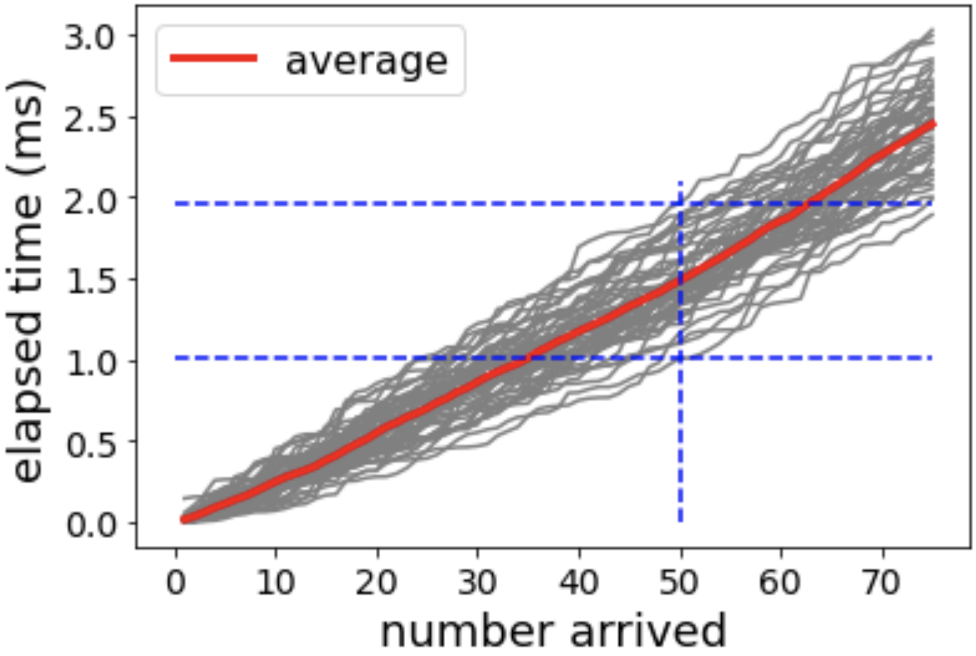

FIG. S9. Time that has elapsed for N particles to arrive within a radius of 4 nm from the center of a disk of outer radius 1250 nm, having started from a uniform distribution of 294 particles in the space between 5 and 1250 nm radius. The grey lines show 50 independent runs, and the thick red line the average over the runs. The vertical and horizontal dashed blue lines show the target (saturation) number of arrivals (50) and upper and lower elapsed times over the 50 runs.

Stage B: Diffusion of C8S3 molecules on the IW and formation of small ordered patches.

When a single C8S3 molecule adsorbs on the IW, it can diffuse on its surface and explore it. In our simulations, we observe that at very low coverage, not all molecules diffuse continuously over the surface, but some appear to be trapped for some time in strongly binding conformations. These are usually associated with one or both aromatic rings lying ‘flat on’ or ‘parallel to’ the surface, as illustrated for both AA and CG models in Fig. S10. This position of the aromatic rings is in contrast to the ‘upright’ or ‘perpendicular’ orientation when molecules are part of small (ordered) patches. Thus, single-molecule mobility on the IW surface appears to be a combination of an activation step (reorientation of the aromatic rings from a ‘parallel’ to ‘perpendicular’ orientation), after which surface diffusion takes place over a time period until a molecules again tightly binds to the surface or joins a patch or domain. MSD curves of diffusing molecules are therefore of relatively short duration and it is difficult to determine proper diffusion coefficients by fitting these curves to

straight lines. Also, experimental diffusion coefficients for these processes are not available.

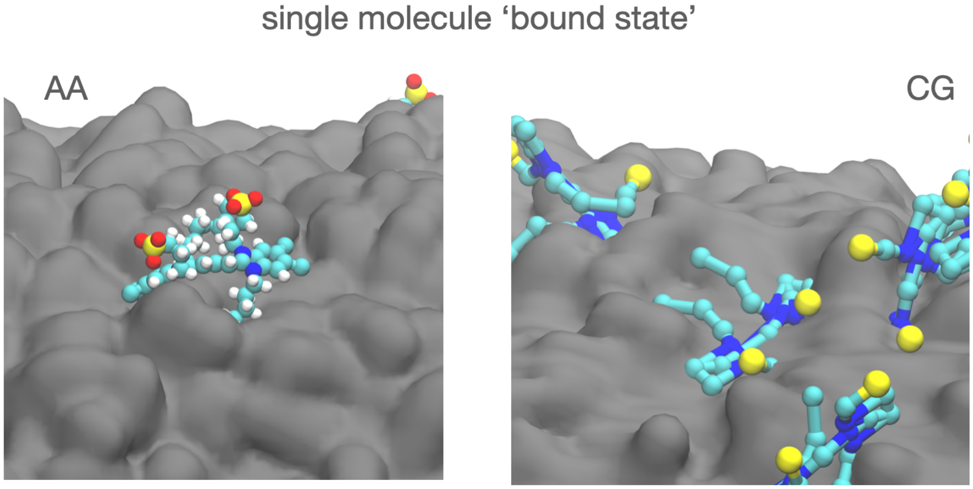

FIG. S10. Representative conformation of relatively long-lived immobilized state of a single C8S3 molecules on the IW surface of a tube.in the atomistic (AA, left) and coarse grain (CG, right) models. Note the parallel orientation of the aromatic cores with respect to the tube surface.

If we calculate the MSD curves and divide them by time, we obtain diffusion coefficients as a running average; if we adopt this method in spite of the 'jump' behavior, we find diffusion coefficients as reported in Table S4. We give the range found for 5 individual molecules. The data in Table S4 show that diffusion of individual molecules on the surface of the tube in the CG MD model is approximately two orders of magnitude slower (10^{-12} m^2s^{-1}) than that in solution ($5 \cdot 10^{-10}$ m^2s^{-1} , see Table S4), and about one order of magnitude larger in the CG model compared to the AA model at 350 K, for which the simulation at 300 K do not give us good enough statistics. Increasing the temperature by 50 K in the CG model increases the diffusion constant by approximately a factor of 6. Note, however, that the spread in the 5 molecules for the lowest temperature (300 K) is particularly large; this probably indicates that an analysis in terms of activated jump processes may reflect the dynamic behavior better.

In the RMSD sense, a single molecule in the CG model will travel $(2Dt)^{1/2} = 1$ nm in 1 μs, the time it takes before the next molecule arrives. This distance is not nearly enough to explore the full length or circumference of the IW, nor the entire length of the 20 nm tube stretch we simulated in the titration simulations, and constitutes a justification for adding more than 1 molecule per 1 μs.

System, temperature, simulation time	D along tube axis $\left(\times 10^{-10}\ \mathrm{m^2s^{-1}}\right)$			D perpendicular to axis $\left(\mathrm{rad^2s^{-1}}\right)$		
	Average	Low	High	Average	Low	High
AA, 350 K, 1 μs	$7 \pm 4\ 10^{-3}$	$1.6\ 10^{-3}$	$1.4\ 10^{-2}$	$7 \pm 3\ 10^{4}$	$2.8\ 10^{4}$	$1.1\ 10^{5}$
CG, 300 K, 4 μs	$1 \pm 1\ 10^{-2}$	$2.8\ 10^{-4}$	$2.3\ 10^{-2}$	$5 \pm 5\ 10^{4}$	$1.2\ 10^{3}$	$1.3\ 10^{5}$
CG, 350 K, 2 μs	$6 \pm 2\ 10^{-2}$	$1.9\ 10^{-2}$	$8.5\ 10^{-2}$	$8 \pm 4\ 10^{5}$	$1.1\ 10^{5}$	$1.3\ 10^{6}$
CG, 400 K, 2 μs	$3 \pm 2\ 10^{-1}$	$8.6\ 10^{-2}$	$5.9\ 10^{-1}$	$4 \pm 2\ 10^{6}$	$1.9\ 10^{6}$	$6.6\ 10^{6}$

TABLE S4. Approximate diffusion coefficients of individual molecules on the OW along the tube axis and perpendicular to the tube axis, based on the motion of 5 individual molecules (see text). Note that 1 on a cylinder of radius 3.5 nm corresponds to $1.23 \cdot 10^{-17}\ \mathrm{m^2s^{-1}}$.

S9. SIMULATED ABSORPTION SPECTRA

In this section, we present the absorption spectra obtained through the multiscale modelling introduced in the main text. First, we show the absorption spectrum of the recovering OW at different times in Fig. S11. The spectra shown are from one of the nine CG MD trajectories available. At 0 μs, the spectrum is dominated by monomers and a few dimers and trimers as indicated by the narrow peak around the monomer energy (20500 $\mathrm{cm^{-1}}$, green dashed). As the OW reassembles, the spectrum is red shifted corresponding to a J-aggregate. The absorption spectrum also becomes broader due to inhomogeneous structural disorder.

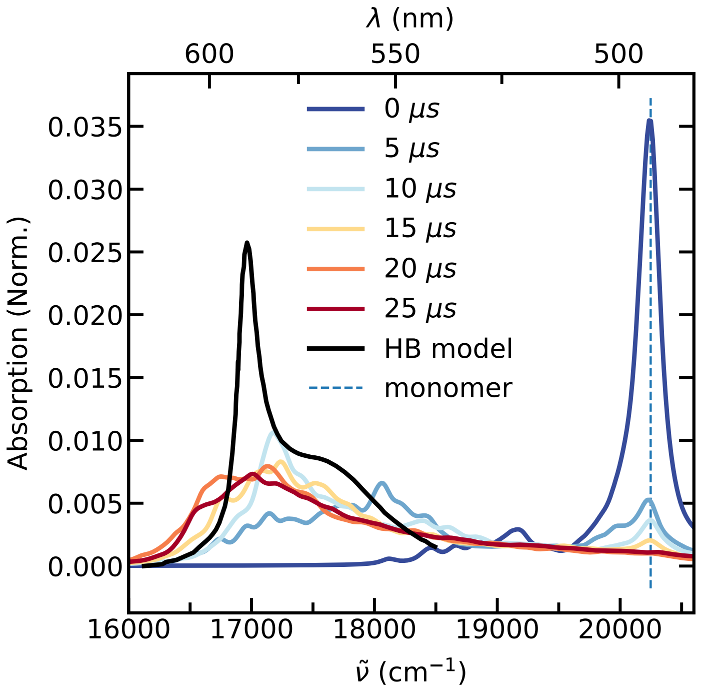

FIG. S11. Simulated absorption spectrum of the recovering OW for different snapshots obtained from a CG MD trajectory. The solid black line corresponds to the OW spectrum obtained by the herringbone (HB) model developed in ref. S8. The vertical green dashed line indicates the monomer frequency of C8S3 molecules. The spectra are normalized with respect to the total intensity.

Using the DBSCAN algorithm (see Methods 4.5) the recovering OW is grouped into several patches. In the recovering OW, domain walls can occur when two patches with different orientations touch each other. There may be resonant excitation energy transfer across the domain walls which in turn will influence the absorption spectrum. To investigate the effect of these domain walls we compare the total absorption spectrum of the recovering OW with the sum of absorption spectra of the individual patches. The sum of absorption spectra of the individual patches is equivalent to neglecting all couplings across domain walls.

The result of the calculation introduced above is shown in Fig. S12. At 0 μs, there are only some dimers present in the simulation box and no substantial patches have formed yet. Hence, there is no difference between the spectrum of patches and the total spectrum. After 10 μs, the OW is partially recovered and the first small patches have formed. As the patches are relatively small in size, the effect of resonant excitation energy transfer between the patches still has a large effect on the overall absorption spectrum. This is observed by the large discrepancy between the

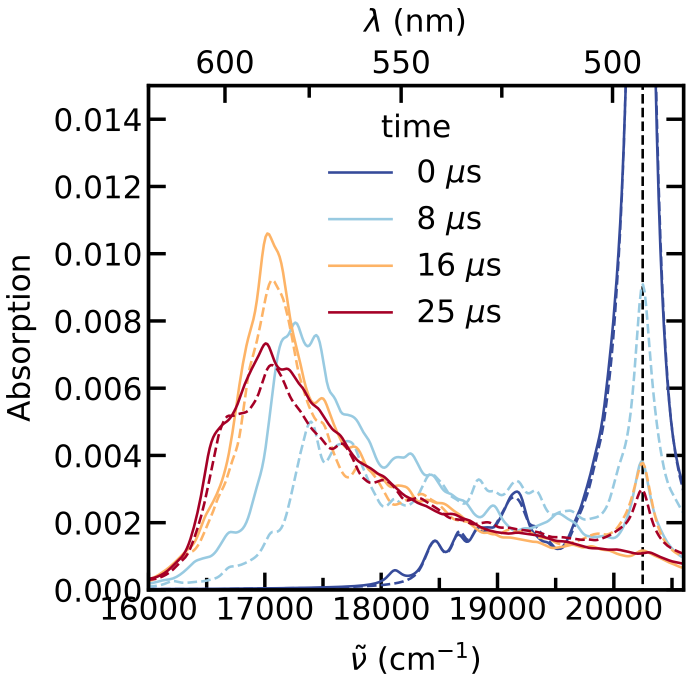

FIG. S12. Comparison of simulated absorption spectra of the entire recovering OW (solid) and the sum of the absorption spectra of individual patches (dashed) for different snapshots of the CG MD trajectory. The vertical dashed black line indicates the monomer energy of C8S3 molecules. The spectra are normalized with respect to the total intensity.

solid and dashed lines. The small domains will eventually merge into several large domains after 20 μs. At this point in time, the effect of domain walls on the absorption spectrum is small. This is explained by the diminishing fraction of molecules located at the edge of a patch as the patch grows larger.

S10. POLARIZATION-RESOLVED 2D SPECTROSCOPY

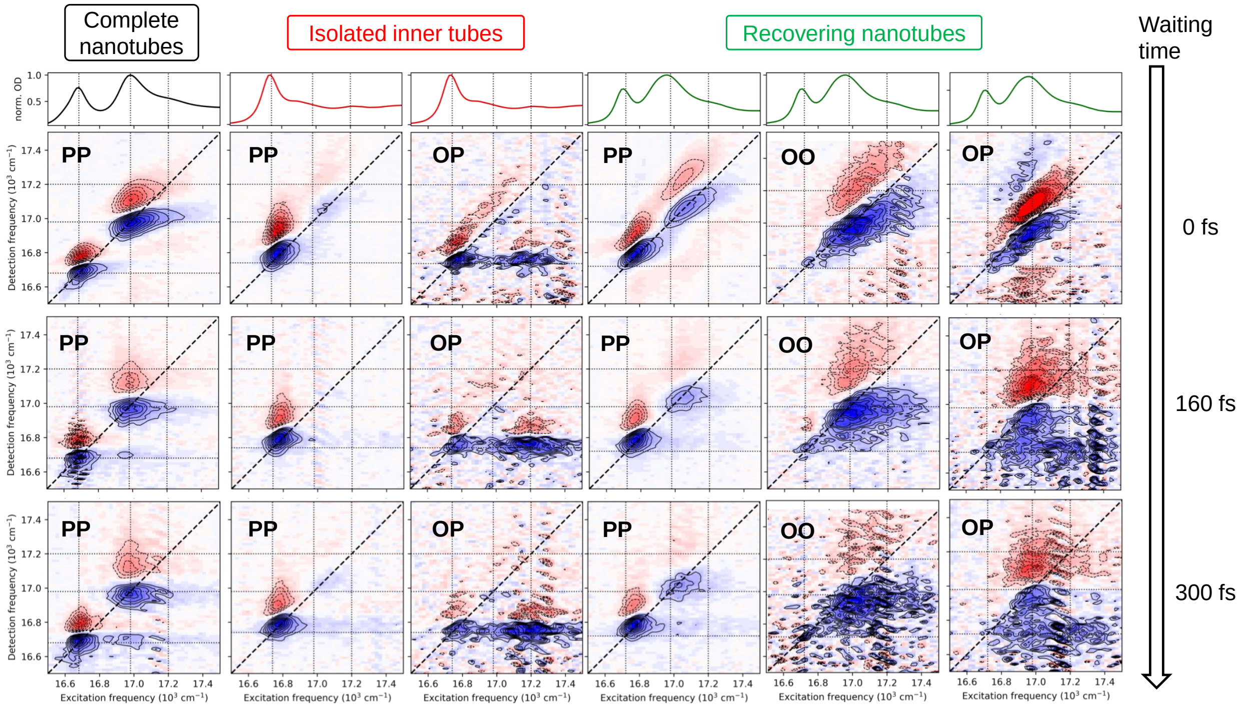

FIG. S13. Polarization-resolved 2D spectroscopy on double-walled nanotubes (first column), isolated inner nanotubes (second and third column), and recovering nanotubes (fourth to sixth column) at three representative waiting times (0 fs, 160 fs, and 300 fs) and for different pump and probe polarizations. The latter are indicated in each panel, where P and O refer to parallel and orthogonal polarization with respect to the flow direction of the sample in the microfluidic channel. The isotropic absorption spectra are shown in the upper panels. In the 2D spectra, the vertical dotted lines indicate the spectral positions of different transitions in the isotropic spectra. Diagonal lines (black, dashed) are drawn at $\tilde{\nu}_1 = \tilde{\nu}_3$, with $\tilde{\nu}_1(\tilde{\nu}_3)$ denoting the excitation (detection) axis of the 2D spectrum. Ground state bleach/stimulated emission (negative) and excited state absorption (positive) signals are depicted with blue and red colours, respectively, and are depicted on a linear colour scale between -1 and $+1$ with colour increments at 0.05. Each spectrum is normalized to the absolute-minimum-signal amplitude, and contour lines is drawn at increments of 0.1 except for the lowest signal level.

In most general terms, the 2D spectra comprises of diagonal peaks ($\tilde{\nu}_1 = \tilde{\nu}_3$) and cross peaks

($\tilde{\nu}_1 \neq \tilde{\nu}_3$), each of which can have positive and negative contributions originating from ground-state bleach/stimulated emission (GSB/SE) and excited state absorption (ESA), respectively, which are depicted with negative (blue colour, Fig. S13) and positive (red colour, Fig. S13) amplitudes to signify the associated change of optical density due to interaction with the pump pulse. Here, use of different polarisation of the pump and probe pulses allows identification of different exciton transitions, and their possible interplay/coupling. As is well-established for molecular J-aggregates, the ESA signal appears spectrally blue-shifted with respect to the GSB/SE signal as a consequence of Pauli repulsion of two excitons[S9,S10].

The 2D spectra of complete nanotubes have been studied intensively[S11–S15] regarding population dynamics, linewidths and spectral broadening as well as exciton dynamics, transfer and annihilation. For complete nanotubes (Fig. S13, first column), the lowest excitonic transitions (i.e., at the bottom of the exciton band belonging to the IW and OW, respectively) are known to be polarised parallel to the nanotubes' long axis. As a result, their amplitude in the 2D spectra can be maximized by setting the pump and probe polarizations both to parallel ('PP') with respect to the flow direction of the sample. The cross peak ($\tilde{\nu}_1 \sim 17000$ cm^{-1}, $\tilde{\nu}_3 \sim 16680$ cm^{-1}) indicating population transfer from the OW to the IW can clearly be distinguished, although its amplitude is still quite low at such short waiting times. In fact, exciton transfer between the OW and the IW has been reported to occur on a time scale of $\sim$ 300 fs [S5,S15–S17]. The horizontal elongation of the OW diagonal peak ($\tilde{\nu}_1 = \tilde{\nu}_3 \sim 17000$ cm^{-1}) as well as the OW to the IW cross peak towards higher frequencies indicate a cross peak associated with one of the higher-lying transitions in the exciton band, which is polarized orthogonally to the NT axis, yet weakly excited here.

After microfluidic flash-dilution (Fig. S13, second and third colummn) the OW diagonal peak ($\tilde{\nu}_1 = \tilde{\nu}_3 \sim 17000$ cm^{-1}) as well as the OW to IW cross peak ($\tilde{\nu}_1 \sim 17000$ cm^{-1}, $\tilde{\nu}_3 \sim 16680$ cm^{-1}) vanishes as expected, while only the IW peak is retained, which undergoes a blue-shift of $\sim$ 60 cm^{-1}. By setting the pump polarization orthogonal to the nanotube orientation, it is possible to suppress the IW diagonal peak ($\tilde{\nu}_1 = \tilde{\nu}_3 \sim 16700$ cm^{-1}) but enhance the amplitude of the cross peak at $\tilde{\nu}_1 \sim 17000$ cm^{-1} and $\tilde{\nu}_3 \sim 16680$ cm^{-1} which is associated with absorption of a higher-lying transition as mentioned in the previous paragraph. The lifetime of intra-band relaxation (following excitation of these higher-lying transitions) to the bottom of the exciton band has previously been reported to be on the order of $\sim$ 60 fs[S17].

The 2D spectra of recovering NT's (Fig. S13, fourth to sixth columns) begin to show the emerging peak at the position of the OW spectra (PP panel). The OO polarization is even more beneficial

for observing the randomly-oriented patches as the regular structures oriented along the flow are not excited nor probed. There are two features worth noticing in the OO-polarized 2D spectra: (i). the spectral width of the diagonal peak of the patches is much broader than that of IW/OW, and (ii). the diagonal elongation is still evident even at 160 fs waiting time. Both signify substantial heterogeneity of the patches which will be explored later is Section S11.

The OP polarization arrangement preferentially excites the disordered structures like patches but probes the response of the regular structures with the dipole moment along the flow. This allows us to obtain the cross peak at $\tilde{\nu}_1 \sim 17000$ cm^{-1} and $\tilde{\nu}_3 \sim 16680$ cm^{-1} developing already at the waiting time of 160 fs. This means that the patches are excited (at $\tilde{\nu}_1$), and within 160 fs the energy is transferred to the IW (at $\tilde{\nu}_3$). Such energy transfer would not have been possible if the patches had been formed in solution, separated from the IW's. Therefore, we conclude that the patches must be attached at the IW surface, in line with cryo-TEM and MD data.

S11. ELLIPTICITY ANALYSIS

The frequency-frequency correlation function shows the rate at which the memory of the initial excitation frequency is lost[S18,S19]. In 2D spectra, this is seen as the disappearance of diagonal elongation of the diagonal peaks: at short waiting times, the peaks are elongated (the memory is still retained) while at longer times the peaks become more round-shaped (the memory is lost). The correlation function allows retrieving time scales of e.g. environmental perturbations, coherence of energy transfer, exciton delocalization etc. Here, we analyzed the 2D peaks at the OW position for the recovering spectra and DWNT's before flash-dilution as a reference.

We evaluated the contributions from homogeneous and inhomogeneous broadening to the 2D spectra by computing the ellipticity function of GSB/SE signal of the OW diagonal peak (as e.g. done in Ref. S18 and Ref. S20). First, the OW peak was selected by cropping the 2D spectrum to the region $\tilde{\nu}_1 = (16900, 17150)$ cm^{-1} and $\tilde{\nu}_3 = (16800, 17400)$ cm^{-1} which contains the positive ESA peak and the negative GSB/SE peak. Second, both peaks were fitted simultaneously with two 45-degree-rotated two-dimensional Gaussian functions given by:

$$\begin{aligned} F(\tilde{\nu}_1, \tilde{\nu}_3) = & -A^{\mathrm{GSB/SE}} \exp\left(-\frac{(\Delta\tilde{\nu}_1^{\mathrm{GSB/SE}} + \Delta\tilde{\nu}_3^{\mathrm{GSB/SE}})^2}{4\sigma_{\mathrm{major}}^2} - \frac{(\Delta\tilde{\nu}_1^{\mathrm{GSB/SE}} - \Delta\tilde{\nu}_3^{\mathrm{GSB/SE}})^2}{4\sigma_{\mathrm{minor}}^2}\right) \\ & + A^{\mathrm{ESA}} \exp\left(-\frac{(\Delta\tilde{\nu}_1^{\mathrm{ESA}} + \Delta\tilde{\nu}_3^{\mathrm{ESA}})^2}{4\tilde{\sigma}_{\mathrm{major}}^2} - \frac{(\Delta\tilde{\nu}_1^{\mathrm{ESA}} - \Delta\tilde{\nu}_3^{\mathrm{ESA}})^2}{4\tilde{\sigma}_{\mathrm{minor}}^2}\right), \end{aligned} \quad \text{(S20)}$$

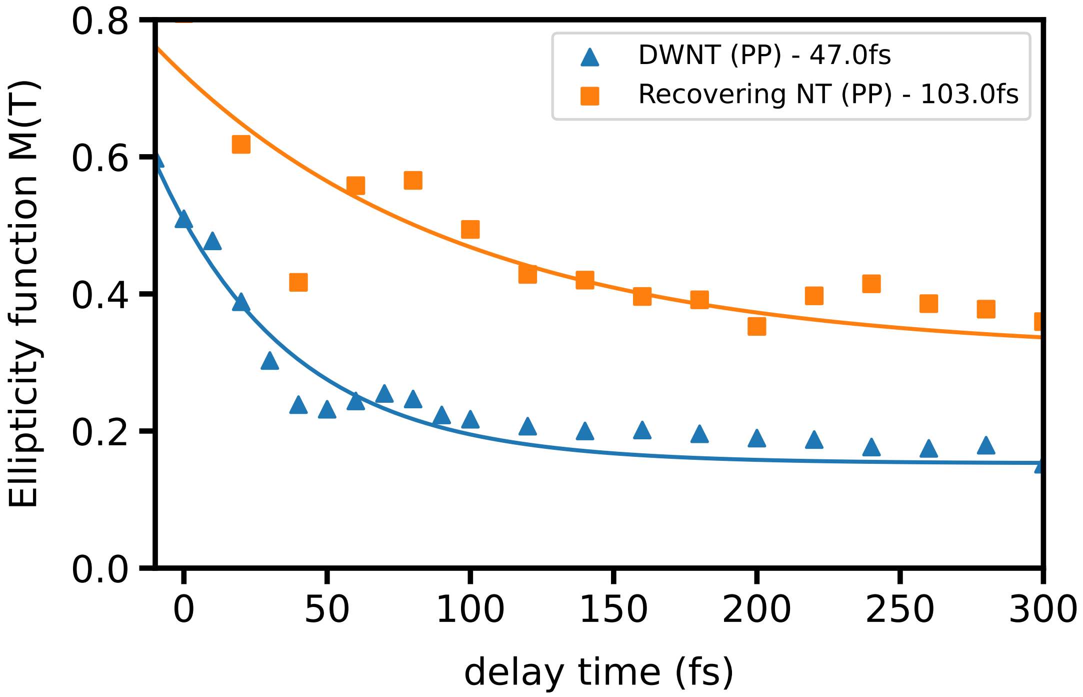

FIG. S14. Comparison of the ellipticity function $\mathrm{M}(T)$ of the OW diagonal peak ($\tilde{\nu}_1 = \tilde{\nu}_3 = 17000$ cm^{-1}) extracted from 2D spectra at different waiting times for DWNT's (blue triangles) and partially recovering NT's (orange squares) measured for PP polarization. The least-squares exponential fit is given by the solid lines with the decay time displayed in the legend.

with $\Delta\tilde{\nu}_1 = \tilde{\nu}_1 - \tilde{\nu}_1^o$ and $\Delta\tilde{\nu}_3 = \tilde{\nu}_3 - \tilde{\nu}_3^o$, where $(\tilde{\nu}_1^o, \tilde{\nu}_3^o)$ is the center of the respective Gaussian and $\sigma^2_{\mathrm{major}}(\sigma^2_{\mathrm{minor}})$ is the standard deviations along the (anti-)diagonal. The standard deviations of the GSB/SE and ESA Gaussians can be different as is indicated by the tilde for the ESA contribution. The additional factor of two in the denominator inside the exponential is due to the 45-degree rotation. Finally, we computed the ellipticity function which is defined as follows[S18]

$$\mathrm{M}(T) = \frac{\sigma^2_{\mathrm{major}} - \sigma^2_{\mathrm{minor}}}{\sigma^2_{\mathrm{major}} + \sigma^2_{\mathrm{minor}}}, \tag{S21}$$

where σ_{major} and σ_{minor} are the standard deviations of the Gaussian fit of the GSB/SE peak in the major and minor directions, respectively.

The ellipticity function $\mathrm{M}(T)$ displayed in Fig. S14 decays fast for complete DWNT's (blue triangle) as the excitons have a large delocalization length[S21]. The recovering NT's (orange squares) show significant slower decay and a substantial offset of $\mathrm{M}(T)$, indicating larger static disorder

and a reduced exciton diffusion. This agrees with the concept of dissolved OW molecules attaching to the IW in patch like structures after flash-dilution, such that the diffusion of the exciton in a patch is restricted by the size of the patch. Therefore, the exciton will not sample all the disorder present in the nanotube, but only the disorder of that patch, yielding an elliptical lineshape (high $\mathrm{M}(T)$). Only at later delay times ($>$ 300 fs), when the exciton moves between patches, will it feel the other disorder environments, and the lineshape of the 2D diagonal peak is more circular (low $\mathrm{M}(T)$). While the effects of static disorder and exciton diffusion cannot be completely disentangled, the increased offset in $\mathrm{M}(T)$ at 300 fs for recovering NT's is a strong indication of increased static disorder with respect to DWNT's. In short, disordered growth in terms of patches makes the ellipticity function decay slower.

REFERENCES

[S1]S. R. Krishnaswamy, I. A. Gabrovski, I. Patmanidis, M. C. A. Stuart, A. H. de Vries, and M. S. Pshenichnikov, "Cryogenic TEM imaging of artificial light harvesting complexes outside equilibrium," Scientific Reports **12**, 5552 (2022).

[S2]J. Madsen and T. Susi, "abTEM: Ab Initio Transmission Electron Microscopy Image Simulation," Microscopy and Microanalysis **26**, 448–450 (2020).

[S3]I. Patmanidis, P. C. T. Souza, S. Sami, R. W. A. Havenith, A. H. de Vries, and S. J. Marrink, "Modelling structural properties of cyanine dye nanotubes at coarse-grained level," Nanoscale Advances **4**, 3033–3042 (2022).

[S4]H. Chen and J.-C. Meiners, "Topologic mixing on a microfluidic chip," Applied Physics Letters **84**, 2193–2195 (2004).

[S5]B. Kriete, J. Lüttig, T. Kunsel, P. Malý, T. L. C. Jansen, J. Knoester, T. Brixner, and M. S. Pshenichnikov, "Interplay between structural hierarchy and exciton diffusion in artificial light harvesting," Nature Communications **10**, 4615 (2019).

[S6]A. Godec and R. Metzler, "First passage time distribution in heterogeneity controlled kinetics: Going beyond the mean first passage time," Scientific Reports **6**, 20349 (2016).

[S7]A. E. Lindsay, R. T. Spoonmore, and J. C. Tzou, "Hybrid asymptotic-numerical approach for estimating first-passage-time densities of the two-dimensional narrow capture problem," Physical Review E **94**, 042418 (2016).

[S8]A. S. Bondarenko, I. Patmanidis, R. Alessandri, P. C. T. Souza, T. L. C. Jansen, A. H.

de Vries, S. J. Marrink, and J. Knoester, "Multiscale modeling of molecular structure and optical properties of complex supramolecular aggregates," Chemical Science (2020), 10.1039/D0SC03110K.

[S9] H. Fidder, J. Knoester, and D. A. Wiersma, "Observation of the one-exciton to two-exciton transition in a J aggregate," The Journal of Chemical Physics **98**, 6564–6566 (1993).

[S10] L. D. Bakalis and J. Knoester, "Pump-Probe Spectroscopy and the Exciton Delocalization Length in Molecular Aggregates," The Journal of Physical Chemistry B **103**, 6620–6628 (1999).

[S11] J. Sperling, A. Nemeth, J. Hauer, D. Abramavicius, S. Mukamel, H. F. Kauffmann, and F. Milota, "Excitons and Disorder in Molecular Nanotubes: A 2D Electronic Spectroscopy Study and First Comparison to a Microscopic Model," The Journal of Physical Chemistry A **114**, 8179–8189 (2010).

[S12] D. Abramavicius, A. Nemeth, F. Milota, J. Sperling, S. Mukamel, and H. F. Kauffmann, "Weak Exciton Scattering in Molecular Nanotubes Revealed by Double-Quantum Two-Dimensional Electronic Spectroscopy," Physical Review Letters **108**, 067401 (2012).

[S13] D. M. Eisele, D. H. Arias, X. Fu, E. A. Bloemsma, C. P. Steiner, R. A. Jensen, P. Rebentrost, H. Eisele, A. Tokmakoff, S. Lloyd, K. A. Nelson, D. Nicastro, J. Knoester, and M. G. Bawendi, "Robust excitons inhabit soft supramolecular nanotubes," Proceedings of the National Academy of Sciences **111**, E3367–E3375 (2014).

[S14] S. Doria, T. S. Sinclair, N. D. Klein, D. I. G. Bennett, C. Chuang, F. S. Freyria, C. P. Steiner, P. Foggi, K. A. Nelson, J. Cao, A. Aspuru-Guzik, S. Lloyd, J. R. Caram, and M. G. Bawendi, "Photochemical Control of Exciton Superradiance in Light-Harvesting Nanotubes," ACS Nano **12**, 4556–4564 (2018).

[S15] R. Augulis, A. Pugžlys, and P. H. M. van Loosdrecht, "Exciton dynamics in molecular aggregates," physica status solidi c **3**, 3400–3403 (2006).

[S16] J. Yuen-Zhou, D. H. Arias, D. M. Eisele, C. P. Steiner, J. J. Krich, M. G. Bawendi, K. A. Nelson, and A. Aspuru-Guzik, "Coherent Exciton Dynamics in Supramolecular Light-Harvesting Nanotubes Revealed by Ultrafast Quantum Process Tomography," ACS Nano **8**, 5527–5534 (2014).

[S17] R. Pandya, R. Y. S. Chen, A. Cheminal, T. Thomas, A. Thampi, A. Tanoh, J. Richter, R. Shivanna, F. Deschler, C. Schnedermann, and A. Rao, "Observation of Vibronic-Coupling-Mediated Energy Transfer in Light-Harvesting Nanotubes Stabilized in a Solid-State Ma-

trix," The Journal of Physical Chemistry Letters **9**, 5604–5611 (2018).

[S18] K. Lazonder, M. S. Pshenichnikov, and D. A. Wiersma, "Easy interpretation of optical two-dimensional correlation spectra," Optics Letters **31**, 3354–3356 (2006).

[S19] K. Okumura, A. Tokmakoff, and Y. Tanimura, "Two-dimensional line-shape analysis of photon-echo signal," Chemical Physics Letters **314**, 488–495 (1999).

[S20] B. Kriete, A. S. Bondarenko, R. Alessandri, I. Patmanidis, V. V. Krasnikov, T. L. C. Jansen, S. J. Marrink, J. Knoester, and M. S. Pshenichnikov, "Molecular versus Excitonic Disorder in Individual Artificial Light-Harvesting Systems," Journal of the American Chemical Society **142**, 18073–18085 (2020).

[S21] B. Kriete, A. S. Bondarenko, R. Alessandri, I. Patmanidis, V. V. Krasnikov, T. L. C. Jansen, S. J. Marrink, J. Knoester, and M. S. Pshenichnikov, "Molecular versus Excitonic Disorder in Individual Artificial Light-Harvesting Systems," Journal of the American Chemical Society **142**, 18073–18085 (2020).
